# 100 plus years of stem cell research—20 years of ISSCR

**DOI:** 10.1016/j.stemcr.2022.04.004

**Published:** 2022-06-14

**Authors:** Urban Lendahl

**Affiliations:** 1 Department of Cell and Molecular Biology, Karolinska Institutet, 17177 Stockholm, Sweden

**Keywords:** stem cell, history, CNS, hematopoiesis, intestine, skin, organoid, blastoid, cell-basedtherapy

## Abstract

The International Society for Stem Cell Research (ISSCR) celebrates its 20^th^ anniversary in 2022. This review looks back at some of the key developments in stem cell research as well as the evolution of the ISSCR as part of that field. Important discoveries from stem cell research are described, and how the improved understanding of basic stem cell biology translates into new clinical therapies and insights into disease mechanisms is discussed. Finally, the birth and growth of ISSCR into a leading stem cell society and a respected voice for ethics, advocacy, education and policy in stem cell research are described.

## Teaser

In this Perspective by Urban Lendahl, the history of stem cell research from discoveries in the late 1800s to the most recent developments is described. The Perspective also discusses the progress in clinical translation of stem cell research discoveries. Finally, the 20-year history of ISSCR as well as the society’s role in research, ethics, advocacy, education, and policy is described.

## Stem cell research—the early years

**Figure 1:**
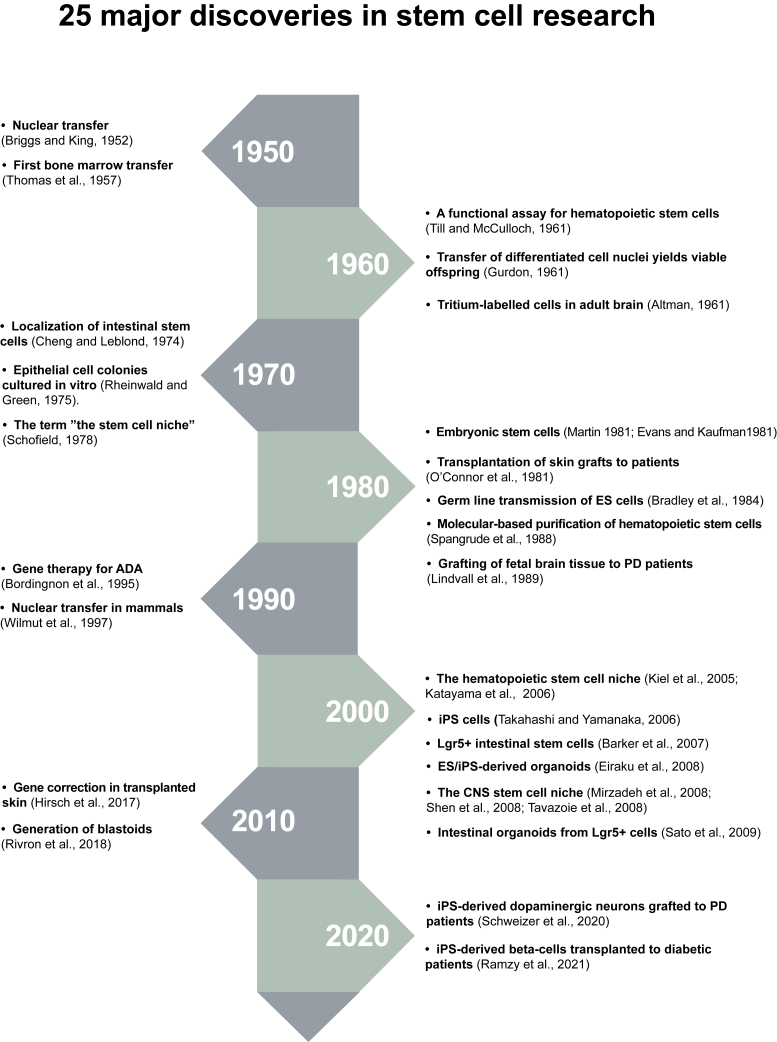
Time axis for 25 major discoveries in stem cell research

Stem cells are defined by the ability to self-renew and to produce differentiated cells, but what could be considered the starting point of this research field? A defining moment is difficult to identify precisely, but an important conceptual prerequisite for stem cell research, and in fact for all cell biology, was the development of the cell theory in the mid-1800s by Rudolf Virchow, Rudolf Remak, and Theodor Schwann and the realization that all cells are derived from other cells through cell division – “*omnis cellula a cellula”* (Virchow, 1858). The first descriptions of the word stem cell also date back to the mid-1800s. Ernst Haeckel used the term “*Stammzellen”* in 1868, but originally in a more phylogenetic context, to denote a unicellular organism from which multicellular organisms developed. In 1877 he extended its use to the fertilized egg, in line with his concept of “ontogeny recapitulates phylogeny” (Haeckel, 1877) (see Figure 1 for a timeline of some of the key discoveries in stem cell research). Theodor Heinrich Boveri and Valentin Häcker used stem cell as a term for cells giving rise to the germ line, thus expanding its use to cell types other than the fertilized egg cell. Häcker also made the important observation that cell division in the crustacean *Cyclops* led to one cell remaining as a stem cell while the other cell differentiated (Haecker, 1892)—an early observation of asymmetric cell division. Boveri characterized cells giving rise to germ cells and somatic cells and referred to them as stem cells (for review see Maehle, 2011). After the initial use of stem cells referring to the germ line, Alexander Maximow, Wera Dantschakoff, and Artur Pappenheim started using the term stem cell in the context of hematopoiesis to denote cells producing the different types of cells in the blood (Dantschakoff, 1908; Maximow, 1909; Pappenheim, 1896).

## Stem cell research milestones—lessons from four organ systems

Progress in stem cell research was reported from many frontiers during the 1900s. In the pre-molecular era, transplantation experiments in amphibians yielded new insights into the role of communication between various organs and tissues for cellular differentiation (Spemann and Mangold, 1924). The development of transgenic technologies, more advanced cell culturing techniques and decoding of genomes from various species further fueled progress in our understanding of stem cells and their various differentiation trajectories. A detailed account of this era is beyond the scope of this review, and I will instead focus on discussing some of the landmark discoveries in stem cell research in four mammalian organs—the hematopoietic system, the skin, the CNS, and the intestine—as each of these organs has provided important concepts for the field at large and revealed opportunities for clinical translation and understanding of disease processes.

### The hematopoietic system

As discussed above, Pappenheim, Maximow, and Dantschkoff introduced the stem cell concept for hematopoiesis, and Pappenheim, in fact, produced a scheme for a hematopoietic stem cell hierarchy not too distant from the version that is agreed upon today (Ramalho-Santos and Willenbring, 2007). It was, however, extensively discussed whether there was a single or more than one type of stem cells for the blood, and there were two camps, dualists and unitarians, with different views on this subject. The unitarian Ernst Neumann observed that hematopoiesis takes place in the bone marrow and suggested that one cell can give rise to all the different blood cells (Neumann, 1868), whereas Paul Ehrlich, for example, advocated separate origins for the different types of cells in the blood (Ramalho-Santos and Willenbring, 2007).

This issue took considerable time to resolve, as hematopoietic stem cells amount to less than 0.01% of all bone marrow cells. An initial landmark discovery in hematopoietic research, and for stem cell research in general, was the first functional assay to quantitate hematopoietic stem cells (or at least closely related multipotent progenitors). This was accomplished by James Till and Ernest McCulloch, who, on the basis of the discovery that bone marrow cells could be successfully transplanted to an irradiated mouse host (Ford et al., 1956), demonstrated that hematopoietic stem cells were endowed with self-renewing as well as multi-lineage differentiation capacities (Becker et al., 1963; Till and McCulloch, 1961). Irving Weissman’s laboratory, building on the Till and McCulloch discoveries, combined the recently invented fluorescence-activated cell sorting (FACS) technology with the use of novel monoclonal antibodies and a negative selection strategy, to provide the first set of molecular markers (Thy1.1^lo^-Sca-1^hi^-Lin^–/lo^) that gave high enrichment of hematopoietic stem cells. As few as 30 such cells were sufficient for 50% survival in transplanted lethally irradiated mice and reconstituted all hematopoietic cell types (Spangrude et al., 1988). Several observations, however, suggested that the Thy1.1^lo^-Sca-1^hi^-Lin^–/lo^ cells remained heterogeneous and that further subdivision of the cells resolved them into long-term self-renewing, short-term self-renewing, and non-self-renewing multipotent progenitors (Morrison and Weissman, 1994). Subsequent work further refined the molecular definition of hematopoietic stem cells (Kiel et al., 2005), but several lines of evidence, including analysis of their epigenetic state and dormancy, suggested that the hematopoietic stem cell pool is molecularly heterogeneous (Foudi et al., 2009; Oguro et al., 2013; Wilson et al., 2008; Yu et al., 2016).

Based on the success in transplanting bone marrow in mice (Ford et al., 1956), it was realized early on that transplantation of hematopoietic stem cells could have huge medical potential, providing an opportunity to replace an ailing or cancerous human hematopoietic system. In 1957, Donnell Thomas and colleagues performed the first allogeneic (from a genetically different individual) bone marrow transplantation in humans (Thomas et al., 1957). Although the first six patients died within 100 days, there were indications of a “take” of the donor bone marrow cells, demonstrating that the concept as such was viable. Georges Mathé a few years later realized that the immunological reaction of the grafted cells toward the cells in the recipient host could be harnessed as a means to help rid the body of remaining cancerous cells, and this controlled graft-versus-host reaction saved patients with relapsing lymphoblastic leukemia that had been transplanted by mixed-donor bone marrow cells (Mathé et al., 1965). The range of diseases that could be treated was gradually extended, and allogeneic transfer was used to replace the hematopoietic system in patients suffering from severe combined immunodeficiency (SCID) and Wiskott-Aldrich syndrome (Bach et al., 1968; Gatti et al., 1968). Following these early pioneering discoveries, further progress in what is now referred to as allogeneic hematopoietic stem cell transfer (allo-HSCT) has been made with regard to immunological matching and immunosuppression, and allo-HSCT is today used routinely in the clinic. To improve the donor base, efforts have been made to establish international registries of unrelated donors, such as the World Marrow Donor Association (Lown et al., 2014). Furthermore, the repertoire of donor cell sources has been expanded to include, for example, umbilical cord blood cells, which, however, take longer to reconstitute the hematopoietic system (see Cieri et al., 2021 for review).

### The CNS

The brain and spinal cord constitute the CNS and arise from stem cells in the embryonic neuroectoderm. Induction of neuroectoderm by the underlying mesoderm was initially demonstrated by Hilde Mangold and Hans Spemann through transplantation experiments in amphibians (Spemann and Mangold, 1924). Retroviral lineage-tracing experiments revealed the existence of embryonic neural stem cells giving rise to both neurons and glial cells (Price and Thurlow, 1988; Turner and Cepko, 1987), and a similar lineage bipotentiality was observed for cultured neural stem cells (Cattaneo and McKay, 1990; Davis and Temple, 1994). A few years later, it was demonstrated that radial glial cells, previously assumed to have more structural roles, served as neural stem cells in the embryonic brain (Malatesta et al., 2000; Noctor et al., 2001). How neural stem cells proceed to acquire specific neuronal identities and the role of transcription factors and morphogens in this process was elucidated by the late Tom Jessell and colleagues (Briscoe et al., 1999; Liem et al., 1997).

The question whether stem cells persisted in the adult brain was a thornier question and a matter of considerable debate for many years. Wilhelm His, in fact, observed cells with mitotic figures near the ventricles of the adult human brain almost 150 years ago (Breunig et al., 2011; His, 1874), supporting that cell division indeed took place. This notion was, however, disputed by many leading contemporary neurobiologists, including Ramón y Cajal, who remained skeptical and argued that “nothing changed after development” (Breunig et al., 2011; Takagi, 2016). In the 1960s, the technique to label dividing cells with tritiated thymidine *in vivo* was developed (Smart and Leblond, 1961). Joseph Altman used this technique to identify cell divisions in the adult rat subventricular zone (SVZ) and dentate gyrus and to show that dividing cells born in the postnatal SVZ migrated along a rostral migratory stream to the olfactory region (Altman, 1961, 1969; Altman and Das, 1965). Adult neurogenesis was not confined only to mammals but was also reported in canary birds, in which new neurons are generated yearly in association with song behavior (Goldman and Nottebohm, 1983). An indication that adult neurogenesis occurs in humans was provided by Fred Gage and the late Peter Eriksson, who identified label-retaining cells in *post mortem* brains from patients who had received bromodeoxyuridine (BrdU) for diagnostic purposes in conjunction with tumor therapy (Eriksson et al., 1998). The precise location of the adult CNS stem cells near the ventricles of the brain was for some time a matter of debate (Doetsch et al., 1999; Johansson et al., 1999), but the current model holds that stem cells with primary cilia (type B cells) give rise to transient-amplifying cells (type C cells), which subsequently become neuroblasts (type A cells). Adult CNS stem cells are largely quiescent (Doetsch et al., 1999), but a certain amount of cellular turnover in the human brain has been demonstrated by Jonas Frisén and colleagues, who developed a unique technique to birth-date cells based on their cellular carbon-14 content, taking advantage of the “spike” in atmospheric carbon-14 levels resulting from above-ground nuclear testing in the early 1960s (Spalding et al., 2005, 2013). The extent to which there is adult neurogenesis in specific human brain regions, such as the dentate gyrus of the hippocampus, is, however, still intensely debated, and reports arguing for (Boldrini et al., 2018; Spalding et al., 2013) or against (Franjic et al., 2022; Sorrells et al., 2018) adult hippocampal neurogenesis have been presented (for review see Kempermann et al., 2018; Paredes et al., 2018).

### Skin

The history of stem cells in the skin is strongly centered around one scientist, Howard Green, who not only pioneered skin transplantation in the clinic but also worked out important cellular principles and trained a cadre of today’s leading epithelial biology scientists. Howard Green started his research in the early 1970s by studying teratomas (as many other scientists did at the time; see below) and in these studies he noted that epithelial cells formed colonies in cell culture and that their ability to expand was enhanced by culturing them on feeder cells (Rheinwald and Green, 1975). Next, he developed procedures for detaching the cultured epithelial sheets (Green et al., 1979) and succeeded in transplanting them to mice (Banks-Schlegel and Green, 1980). In a landmark study, Green transplanted two patients with third-degree burn injuries with autologous skin grafts (O’Connor et al., 1981), which represented the first step toward a life-saving therapy for patients with severe burn injuries. Skin transplantation is nowadays well established in the clinic and has more recently been combined with gene correction techniques: a patient with junctional epidermolysis bullosa was grafted with skin in which the *LAMB3* gene was inserted to provide correct expression of laminin-332 (Hirsch et al., 2017).

Skin constitutes approximately 15% of body weight and is composed of two layers (epidermis and dermis), as well as hair follicles, sweat glands, and sebaceous glands. Epidermal stem cells are located in the basal layer (Barrandon and Green, 1987) and balance self-renewal and production of keratinocytes that progress through the upper, suprabasal layers, eventually ending up as dead squames, which are shed from the stratum corneum (for review see Blanpain and Fuchs, 2009). Differentiation is accompanied by a coordinated change in keratin expression to appropriately adapt the cytoskeleton of the keratinocytes to their position in the epidermis (Fuchs and Green, 1978, 1980). The choice between self-renewal and differentiation of the epidermal stem cells is, at least in part, controlled by asymmetric cell division and the angle of the cleavage plane (Lechler and Fuchs, 2005; Smart, 1970). Regulation of clone size and distribution of clones derived from individual epidermal stem cells and whether clone expansion involves an intermediate transient-amplifying cell population are topics that have been intensely studied in the mouse. Models based on stochastic events, neutral drift, or distinct stem cell pools and lineages have been proposed (Clayton et al., 2007; Gomez et al., 2013; Jones and Watt, 1993; Mascré et al., 2012; for review see Rognoni and Watt, 2018).

In addition to the stem cells residing in the basal layer, the hair follicles contain molecularly distinct stem cell populations positioned at different locations of the hair follicle, including the bulge region and the sebaceous gland (Cotsarelis et al., 1990; Jensen et al., 2009; Tumbar et al., 2004). While bulge stem cells normally give rise only to the hair follicle, lineage tracing and transplantation experiments revealed that they can contribute to both hair and epidermis (Blanpain et al., 2004; Claudinot et al., 2005). A specific feature of the hair follicle stem cells is that they need to tune their activity to the hair cycle, which switches between a resting (telogen), a regenerative (anagen), and a destructive (catagen) phase (Blanpain and Fuchs, 2009). Fgf18 and BMP6 produced from differentiating cell progeny play key roles for maintaining quiescence (Hsu et al., 2011), while SHH produced from transient-amplifying cells activates the stem cells (Hsu et al., 2014). In the normal, non-injured state, the various stem cell populations give rise to distinct subsets of differentiated cells (Jensen et al., 2009; Page et al., 2013), but in response to injury, all stem cell populations give rise to epidermal cells (Aragona et al., 2017; Donati et al., 2017; Ge et al., 2017; Park et al., 2017). This “all hands on deck” stem cell contribution to epidermal cells upon injury is likely important to rapidly heal an epidermal wound. Interestingly, the response of epidermal stem cells may be tuned by previous experiences, such as acute inflammation (Naik et al., 2017).

### Intestine

The intestine is a hostile environment for cells, and in line with this, there is a very rapid cell turnover, with an average life span of only a few days for intestinal cells. The intestine is composed of two principal categories of cells: absorptive (enterocytes and M cells) and secretory (goblet, Paneth, tuft, and enteroendocrine cells) cells, and both categories originate from intestinal stem cells (Beumer and Clevers, 2021). The tiny crypt-base columnar (CBC) cells, localized at the bottom of the intestinal crypts between the much larger Paneth cells, were first proposed as the enigmatic crypt stem cell (Cheng and Leblond, 1974). Later, Chris Potten proposed that cells located at position +4 (counting from the crypt base) rather than the CBC cells constituted the “real” stem cell (Potten et al., 2002), which remained the dominant view for years. In an early version of genetic lineage tracing, cell progeny born at or near the crypt base were demonstrated to migrate away from the crypts along the crypt-villus axis (Winton et al., 1988). The discovery that the crypt-base columnar cells express Lgr5 provided definitive, lineage-tracing-based evidence in support of CBCs as the crypt stem cell (Barker et al., 2007). An interesting feature of the Lgr5^+^ intestinal stem cells is that they constantly divide and show high telomerase activity (Sato et al., 2009; Schepers et al., 2011), which sets them apart from quiescent stem cells in many other organs. Lineage tracing of the intestinal stem cells by virtue of their Lgr5 expression has provided insights into clonal distribution of differentiated cell progeny (Barker et al., 2007), and neutral drift and competition between the stem cells eventually leads to clonality in the crypts (Lopez-Garcia et al., 2010; Snippert et al., 2010). More recently, a slow-dividing Lgr5^+^ cell population has been identified, which normally gives rise to Paneth and enteroendocrine cells, but upon injury can generate all intestinal cell types (Buczacki et al., 2013). An intriguing finding is that more differentiated intestinal cell types can revert to become intestinal stem cells upon injury (van Es et al., 2012; Jadhav et al., 2017; Tetteh et al., 2016), thus helping to replenish the intestinal stem cell pool.

## The stem cell niche

Stem cells do not function in splendid isolation; they are, in fact, highly dependent on interactions with surrounding cells and tissues, which constitute the stem cell niche. Ray Schofield launched the concept of a stem cell “niche” in the hematopoietic system (Schofield, 1978), and considerable progress has since then been made in terms of characterizing the hematopoietic stem cell niche. In the 1970s, Michael Dexter and colleagues showed that stromal cells were important for culturing of hematopoietic stem cells *in vitro* (Dexter et al., 1977). Next, osteoblasts in the bone marrow were considered to be the important niche cells (Calvi et al., 2003; Zhang et al., 2003), but results from subsequent studies have instead revealed that hematopoietic stem cells reside near sinusoidal blood vessels in the bone marrow (Kiel et al., 2005, 2007; Sugiyama et al., 2006), suggesting that the endothelial or paravascular cells provide the important niche signals and that the osteoblasts exert more indirect effects. Indeed, leptin receptor-positive stromal cells, together with endothelial cells, produce factors such as stem cell factor (SCF) and Cxcl12, which are critical for stem cell maintenance (Ding and Morrison, 2013; Ding et al., 2012; for review see Comazzetto et al., 2021; Morrison and Scadden, 2014). Furthermore, the late Paul Frenette demonstrated that the sympathetic nervous systems provided signals for hematopoietic stem cell mobilization (Katayama et al., 2006). Hematopoietic stem cell maintenance is also influenced by various types of immune cells, such as granulocytes and monocytes, located at specific sites in the bone marrow (Hérault et al., 2017; Zhang et al., 2021), as well as by stress conditions (Severe et al., 2019), offering mechanisms by which changes in overall physiological status can be sensed and influence hematopoietic stem cell activity.

In the adult brain, stem cells are primarily located in the subventricular zone and the hippocampus, and progress has been made in decoding their niches. In the subventricular zone, the stem cells reside in pinwheel-like niche structures near the brain vasculature and cerebrospinal fluid (Mirzadeh et al., 2008; Shen et al., 2008; Tavazoie et al., 2008), while neural stem cells in the hippocampus are positioned close to the inner granule cell layer in the dentate gyrus (Sun et al., 2015). How changes in niche composition, for example with regard to nutrient sensing, contributes to the cognitive decline observed during aging is an emerging research area (for review see Navarro Negredo et al., 2020). Other, somewhat less intuitive, and longer-range niche components in the brain are the meninges, which are membranous structures circumscribing the brain, and the choroid plexus, the major source of cerebrospinal fluid production. Both the meninges and the choroid plexus produce factors that influence neural stem cells, such as CCL2, CXCL12, and retinoic acid (Belmadani et al., 2015; Radakovits et al., 2009; Siegenthaler et al., 2009; Silva-Vargas et al., 2016). The microenvironment for oligodendrocyte progenitor cells has been shown to stiffen with age in the brain, which contributes to an age-related decline in oligodendrocyte production (Segel et al., 2019).

In the skin, the epidermal stem cells in fact contribute to shaping their own niche by producing the extracellular matrix on which they sit in the basal layer (Blanpain and Fuchs, 2009). The extracellular matrix also provides a reference point for the stem cell division plane, dictating the balance between self-renewal and differentiation (see above). External stimuli can affect the niche to regulate epidermal stem cell activity, and such stimuli include mechanical stretching of the skin (Aragona et al., 2020; Fiore et al., 2020), as well as stress and activity in the sympathetic innervation to the skin (Shwartz et al., 2020; Zhang et al., 2020). Furthermore, alterations in the epidermal stem cell secretome modulate surrounding lymphatic capillaries, which constitute part of the epidermal stem cell niche (Gur-Cohen et al., 2019). For hair follicle stem cells, the dermal papilla, located adjacent to the bottom of the hair follicle, is a driver of stem cell activation, and immune cells (T cells and macrophages) also influence hair follicle stem cells (Ali et al., 2017; Castellana et al., 2014).

Paneth cells, i.e., the cells intercalated between the Lgr5^+^ stem cells, constitute an important niche component for intestinal stem cells (Sato et al., 2011). If Paneth cells are experimentally ablated, they can be replaced by other cells, such as enteroendocrine cells, that take over the niche function (van Es et al., 2019; for review see McCarthy et al., 2020). Paneth cell replacement, along with replacement of ailing or lost intestinal stem cells by more differentiated cells (see above), provides important mechanisms for safeguarding intestinal cell turnover and contributes to making the intestine quite resilient to injury. More “long-range” niche signals provided to the intestinal stem cells have also been identified, with mesenchymal cells (a.k.a. myoepithelial cells or telocytes) in the vicinity of the crypts and villi providing secreted factors such as Wnt, R-spondin, and BMP-inhibitors (Beumer and Clevers, 2021). Analysis of stem cell niches in different organs is a very active research field, and further progress is expected regarding the response of the niches to altered physiological conditions, injury, and age, and to shed light on how stem cells themselves contribute to the niche (Fuchs and Blau, 2020; Gola and Fuchs, 2021).

## The quest for cellular rejuvenation and pluripotency

In addition to understanding the underpinning mechanisms of stem cell maintenance and differentiation, there was a parallel interest in learning whether the phenotype of a differentiated cell could in some way be reversed, leading to “rejuvenation” of a mature cell. This was first explored by asking whether a differentiated cell nucleus could revert to a more immature state if transferred to an enucleated, undifferentiated cell. The idea of nuclear transfer was already contemplated by Hans Spemann, but it was Robert Briggs and Thomas King who showed that such an experiment was technically possible, by demonstrating that nuclei from frog blastula transplanted into enucleated frog eggs gave rise to tadpoles (Briggs and King, 1952). John Gurdon, using the Briggs and King somatic cell nuclear transfer (SCNT) technology, then provided the first demonstration that tadpoles could be produced after transplantation of a cell nucleus from a differentiated adult frog cell (Gurdon, 1962). Following this pioneering report, SCNT was established in mammalian species, including sheep (Wilmut et al., 1997) and mice, where a combination of SCNT and gene therapy could correct a genetic defect in the nuclear donor strain (Rideout et al., 2002). SCNT has also found novel medical uses, for example in the mitochondrial replacement technique (MRT), an emerging strategy to correct mitochondrial disease in humans. MRT rests on a combination of *in vitro* fertilization techniques originally pioneered by Patrick Steptoe and Robert Edwards (Steptoe et al., 1971) and SNCT and is used as a means for mothers carrying severe mtDNA mutations to have genetically related children (Cohen et al., 2020). Technically, by transferring the male and female pronuclei from a fertilized egg from parents with mitochondrial disease by pronuclear DNA transfer (PNT) into an enucleated donor zygote, or alternatively transferring the metaphase II spindle complex from the mother’s oocyte into an enucleated donor oocyte, the faulty mitochondria from the mother are replaced with those from the donor zygote or oocyte (see also below under the ISSCR guidelines).

To learn to culture the most undifferentiated cells *in vitro* represented another Holy Grail for stem cell research, as it was expected to give insights into cellular pluripotency and how such a state could be maintained. It was argued that pluripotent cells should reside in the inner cell mass of the blastocyst but possibly also in teratomas and teratocarcinomas, tumors containing a bewildering variety of differentiated cell types and tissue, suggesting the existence of highly undifferentiated stem cells in these tumors (for review see Solter, 2006). Leroy Stevens and Clarence Cook Little showed that the propensity for developing testicular teratomas, which normally is very low in mice, was elevated in a specific mouse strain, the 129-strain (Stevens and Little, 1954). This opened new vistas for gaining insights into this tumor type, and teratocarcinomas from the 129-strain could be propagated in the abdominal cavity of mice (Kleinsmith and Pierce, 1964). Subcutaneous transplantation of single cells from the ascites fluid contributed to a variety of tissues (Kleinsmith and Pierce, 1964), revealing that multipotent cells (referred to as embryonal carcinoma cells) could be identified experimentally. Ralph Brinster advanced the transplantation paradigm further by demonstrating that transfer of embryonal carcinomas cells into the early mouse blastocyst resulted in chimeric offspring (Brinster, 1974).

The question, however, remained whether cells that could give rise to all cell types in an animal, i.e., totally pluripotent cells, could be identified and harnessed *in vitro*. Gail Martin and Martin Evans managed to culture inner-cell mass-derived cells (referred to as embryonic stem [ES] cells) and produce teratocarcinomas upon transplantation (Evans and Kaufman, 1981; Martin, 1981). Three years later, Evans and colleagues demonstrated that ES cells could yield germline chimeric mice (Bradley et al., 1984). ES cell lines were next generated from non-human primates (Thomson et al., 1995) and humans (Shamblott et al., 1998; Thomson et al., 1998).

The SCNT experiments discussed above demonstrated that a differentiated cell nucleus could be rejuvenated if placed in an appropriate juvenile cellular environment; but would it be possible to revert an intact differentiated cell into an undifferentiated, pluripotent state? For some time, this remained more of a thought experiment, and the route to inducing pluripotency in a cell was viewed to be very complex, if not impossible. The molecular decoding of ES cells, however, provided insights into factors required to maintain the pluripotent state in the culture dish (Chambers et al., 2003; Nichols et al., 1998). This information was used to test combinations of such factors, and the discovery that expression of only a very small set of transcriptional regulators (Oct4, Sox2, cMyc, and Klf4) was sufficient to convert a differentiated mouse fibroblast into an induced pluripotent stem cell (iPS cell) came as a surprise to the field (Takahashi and Yamanaka, 2006). The generation of human iPS cells was published a year later (Takahashi et al., 2007; Yu et al., 2007). It was also soon demonstrated that provision of a small set of transcriptional regulators could drive a direct conversion of one type of differentiated cell into another, without proceeding through the pluripotent state. In this way, β-cells, oligodendrocytes, and neurons were produced from other differentiated cell types by direct lineage conversion (Vierbuchen et al., 2010; Yang et al., 2013; Zhou et al., 2008; for review see Falk et al., 2021). The notion that a direct lineage conversion could be obtained by expression of specific combinations of transcription factors was also in line with a classical observation by the late Harold Weintraub that expression of MyoD was sufficient to convert fibroblasts (10T1/2 cells) into myoblasts (Lassar et al., 1986).

## Organoids

Protocols were developed to steer ES and iPS cell differentiation toward specific differentiated cell fates, and when combined with the introduction of disease-specific mutations into the genome of the pluripotent cells, new light was shed on the molecular basis for monogenic diseases, such as amyotrophic lateral sclerosis, Parkinson’s disease, and long QT syndrome (see Soldner and Jaenisch, 2018 for review). Most of the early protocols, however, relied on culturing the cells as a flat two-dimensional (2D) monolayer, and it made intuitive sense that three-dimensional (3D) culturing of cells would more closely recapitulate the *in vivo* situation and thus be superior to 2D culturing. There was, therefore, an interest in exploring whether ES and iPS cells could be not only differentiated but guided toward forming more complex mini-organs, called organoids, when cultured in 3D (see Lancaster and Knoblich, 2014 for review). When ES or iPS cells were allowed to proceed through an embryoid body-like state, recapitulating early embryo development, they revealed signs of self-organization into organ-like structures. Pioneering research by the late Yoshiki Sasai yielded retinal and brain organoids (Eiraku et al., 2008, 2011), and subsequent work by Jürgen Knoblich’s and Sasai’s research groups demonstrated that brain organoids with advanced anatomical organization could be generated and that brain disease-specific features could be mimicked in the organoids (Kadoshima et al., 2014; Lancaster et al., 2013).

Adult-tissue stem cells from various epithelial structures turned out to be an alternative cellular source for organoid generation. A breakthrough in this area was the discovery that intestinal Lgr5^+^ stem cells (see above) gave rise to organoids with many features of the intestinal crypt (Sato et al., 2009) and that such organoids engrafted successfully when transplanted to the mouse intestine (Yui et al., 2012). Organ-specific organoids have now been generated from Lgr5^+^ stem cells from most other organs, including liver (Huch et al., 2013). Organoids not only shed light on principles for organ generation but are increasingly used to study disease mechanisms, for example in infectious disease research, where the effects of Zika virus exposure have been studied in brain organoids (Qian et al., 2016), and various organoid systems have rapidly been adapted for COVID-19 research (see Geurts et al., 2021 for review). The effects of specific disease mutations have been explored in organoid systems, including mutations causing microcephaly (Lancaster et al., 2013), CFTR (Dekkers et al., 2013) and liver diseases such as alpha1-antitrypsin and Alagille syndrome (Huch et al., 2015). Although most examples of disease modeling in organoids still come from monogenic diseases, organoids from patients with genetically more complex, non-monogenic diseases, such as biliary atresia, have also unveiled disease-specific phenotypes (Babu et al., 2020). Finally, organoids are increasingly used to unravel the molecular basis for various types of cancer and to explore personalized-medicine approaches for cancer therapy (Kastner et al., 2021). As will be discussed later, we can envisage the generation of increasingly more complex organoid systems, and analysis of interactions between organoids and specific cell types, such as immune cells. To grow larger organoids will at some point likely require vascularization, and blood vessel organoids have recently been generated (Wimmer et al., 2019).

An interesting recent offshoot from the organoid tree is the development of blastoids, blastocyst-like structures that have spurred a new research field referred to as synthetic embryology (Li et al., 2022). Blastoids were first produced from mouse cells, where an assembly of ES cells and trophoblast stem cells produced blastocyst-like structures (Rivron et al., 2018). More recently, human blastoids have been developed that undergo lineage specification in the order expected of blastocysts and that can attach to endometrial cells (Kagawa et al., 2022; Yanagida et al., 2021; Yu et al., 2021).

## Toward clinical translation of stem cell research

Given the vast body of basic stem cell research, how rapid is the transition of this information to clinical applications in the different organ systems? Progress is made on many fronts, but the extent of clinical translation differs between organ systems. Allo-HSCT from bone marrow, peripheral blood, or cord blood is well established and currently used routinely in the clinic to treat hematological malignancies or congenital immunodeficiencies such as *β*-thalassemia, Fanconi anemia, sickle cell anemia, acute and chronic leukemia, non-Hodgkin’s lymphoma, and Hodgkin’s disease (Ferrari et al., 2021). There is also considerable progress in developing gene correction strategies for autologous hematopoietic stem/progenitor cells. Adenosine deaminase deficiency (ADA), an immune-deficiency disorder, was cured by adding the ADA gene into a patient’s bone marrow cells and peripheral blood lymphocytes, providing long-term immune system restoration (Bordignon et al., 1995). However, the use of retroviral vectors for gene corrections in hematopoietic cells initially resulted in aberrant viral integrations leading to T cell acute lymphoblastic leukemia (Howe et al., 2008), For a long time, the risk of tumor development cast a shadow over the entire gene therapy field, but improvement in viral vectors, for example through the use of self-inactivating gammaretroviral or lentiviral vectors, have enhanced efficacy and led to safer therapies (Ferrari et al., 2021). Direct gene editing, rather than virus-based gene insertions, is an interesting avenue to explore, and successful correction of the mutated *IL2RG* gene in hematopoietic stem cells from a SCID patient has been reported (Genovese et al., 2014). CRISPR-Cas9 gene modification strategies are being developed but are not yet clinically approved (Ferrari et al., 2021).

In contrast to skin transplantation and allo-HSCT, where therapies are well established in the clinic, cell replacement therapies for brain diseases are still a work in progress. Parkinson’s disease (PD) has long been an attractive candidate for stem cell therapy because a specific cell type, the A9 nigral neurons providing dopaminergic innervation to striatum, are lost in PD. Furthermore, the efficacy of dopaminergic agents such as L-DOPA and levodopa declines after a few years and can cause side effects, notably dyskinesia (involuntary movements). Important steps toward PD cell therapy included proof-of-principle for survival of dopaminergic neuronal grafts in rats (Olson and Seiger, 1973) and development of a PD-mimicking rat model where dopaminergic neurons in the nigrostriatal system were chemically depleted by 6-hydroxydopamine (6-OHDA) (Ungerstedt, 1968). It was next reported that transplantation of fetal dopaminergic grafts improved outcome in the rat 6-OHDA model (Björklund et al., 1980; Freed et al., 1981; Perlow et al., 1979). A study using adrenal medullary tissue grafted into two PD patients (Backlund et al., 1985) spurred open-label trials of human fetal ventral mesencephalic allografts (Lindvall et al., 1989), which showed some evidence of clinical success and cell survival, based on PET imaging. This was followed by a series of further open-label studies and then two double-blind NIH-funded placebo-controlled studies in the US, which gave conflicting results regarding the extent of improvement, if any, that was seen in transplanted patients. The analysis was also complicated by inclusion of patients with different disease severity, the use of differing amounts of the transplanted tissue, and different levels of immunosuppression (Barker et al., 2015). It was also noted that Lewy body formation was observed in some of the grafts (Kordower et al., 2008; Li et al., 2008), indicating that the pathology may spread from host tissue to the graft.

The finding that some PD patients having received fetal grafts showed some long-term improvements (Kefalopoulou et al., 2014) was encouraging, but the use of fetal tissue is ethically problematic, and the supply of tissue is limited. Therefore, *in vitro* differentiation of ES and iPS cells along the dopaminergic neuron trajectory has been intensely pursued as an alternative source of transplantable cells. Protocols for differentiation of dopaminergic, tyrosine hydroxylase-positive neurons were established by several groups, and the realization that dopaminergic neurons were derived from floor plate cells (Bonilla et al., 2008; Ono et al., 2007) led to considerably improved protocols and differentiation efficiency (Chambers et al., 2009; Kriks et al., 2011) as well as successful outcomes on transplantation of the resulting cells into animal models (Kikuchi et al., 2017; Kirkeby et al., 2017; see Kim et al., 2020 for review).

With the data from fetal and ES/iPS transplantations at hand, clinical translation is now pursued in different projects and consortia, including GForce-PD, an international consortium to advance and harmonize stem cell-derived dopamine cell transplant therapies for PD (Barker et al., 2015). The first PD patients have recently been grafted with iPS-derived neurons (Schweitzer et al., 2020; Takahashi, 2020; see also Kim et al., 2020; Parmar and Björklund, 2020 for review). Transplantations have recently started or will soon start also at other centers and consortia, including a Cambridge-Lund study (STEM-PD), Kyoto University (Takahashi, 2020), and Sloan Kettering (Kim et al., 2020). It will be interesting to learn how effective stem cell-based therapies will be for PD patients across the various consortia and how such therapies will compare with other therapies for advanced PD, such as deep brain stimulation (DBS) (Barker et al., 2021).

Transplantation of different retinal cell types is an interesting avenue to restore vision for patients with certain eye diseases, such as dry age-related macular degeneration (AMD). AMD is accompanied by progressive loss of retinal pigment epithelial (RPE) cells, which are crucial for survival of the overlying photoreceptor cells. As is the case for PD, fetal transplants have been performed to replace lost cells, paving the way for stem cell-based approaches (Algvere et al., 1997). That study also highlighted the importance of immunosuppression despite the fact that the eye is considered somewhat immunoprivileged. Robust xeno-free, defined, and scalable differentiation protocols for RPE and photoreceptor cells have been developed and have shown promise in animal models (Osakada et al., 2008; Vaajasaari et al., 2011; McGill et al., 2017; Plaza Reyes et al., 2020; Ribeiro et al., 2021). The first transplantation of ES cell-derived RPE cells in humans was carried out in 2012 in patients with dry AMD and Stargardt disease, a disease leading to macular degeneration in younger individuals (Schwartz et al., 2012). In a subsequent larger study, there was visual improvement in half of the patients but also some significant side effects, such as cataract and inflammation (Schwartz et al., 2015). The first autologous iPSC-based cell therapy trial for any disease was performed in 2014 aiming to treat neovascular AMD (Mandai et al., 2017) and was later followed by an alternative strategy with banked HLA-matched allogeneic iPSCs to reduce the need of immunosuppression (Sugita et al., 2020). An alternative strategy to minimize immunological rejection has recently been reported through removal of HLA class I and II (Petrus-Reurer et al., 2020). Although transplantation of PSC-derived RPE cells shows promise for potentially halting further progression of disease, restoration of vision will ultimately also require replacement of lost photoreceptors.

In type 1 diabetes, β-cells in the pancreas are lost, and although whole pancreas or islet transplantation provides relief from hypoglycemia, donor tissue is in limited supply (Krentz et al., 2021), making type 1 diabetes a candidate for cell therapy-based approaches. A first-generation protocol for ES or iPS cell differentiation to β-cells initially yielded mixed cellular phenotypes and no glucose-responding cells (D’Amour et al., 2005). Advanced protocols produced human β-cells that turned out to be glucose responding and insulin secreting but only after transplantation and further maturation in mice (Kroon et al., 2008). The company ViaCyte conducted a phase 1/2 clinical trial using these immature endocrine cells held in an encapsulation device, and although the cells were tolerated after transplantation, no evidence of insulin production was reported (Henry et al., 2018). The next step was to create holes in the encapsulation device to allow nutrients and oxygen exchange, and with this modification, now requiring the administration of immunosuppressants, a few of the 15 patients receiving the cells and device have shown evidence of insulin production (stimulated C-peptide levels) (Ramzy et al., 2021). The company Vertex, after having acquired Semma therapeutics, has taken a different approach. By use of stem cell-derived islets that are fully differentiated and mature (Pagliuca et al., 2014), positive results in blood glucose control and therapeutic levels of insulin production have been reported from the first patient transplanted with such cells (VX880) into the liver, again along with immunosuppressants.

Myocardial infarction leads to muscle loss and formation of fibrotic tissue, and there are currently no functional therapies to replace lost or ailing cardiomyocytes. Moreover, the heart appears to be one of the organs lacking a robust endogenous adult stem cell pool (Senyo et al., 2013). For two decades, various types of adult cells have therefore been transplanted to improve post-infarction heart function, but with rather modest success (see Murry and MacLellan, 2020 for review). Hopes have since been pinned instead on cell therapy using *in vitro*-engineered cardiomyocytes. Initially, it may have been thought that development of such therapies would be rather straightforward, given that ES cells easily could be differentiated into beating cardiomyocytes in the culture dish, an *in vitro* differentiation paradigm that has been used for disease-modeling of different channelopathies (Giacomelli et al., 2020). The *in vitro*-differentiated cardiomyocytes are, however, thus far immature and do not exhibit all the properties of a fully differentiated cardiomyocyte (Karbassi et al., 2020). This has hampered clinical testing, but engraftment in animal models provides reason for cautious optimism. Survival and engraftment of cardiomyocytes have been demonstrated in rats and guinea pigs (Riegler et al., 2015; Weinberger et al., 2016), and improved cardiac function following transplantation has been demonstrated in experimentally infarcted macaques (Liu et al., 2018) and pigs (Romagnuolo et al., 2019). Furthermore, microvascular grafts have been tested and shown to improve perfusion in infarcted rat hearts (Redd et al., 2019), and patches of iPS-derived cardiomyocytes, smooth muscle cells, and endothelial cells to experimentally infarcted pig hearts resulted in improved ventricular function (Gao et al., 2018). The transplantation of ES/iPS-derived cardiomyocytes to pigs and non-human primates has however also caused arrythmias, presumably as a consequence of the transplanted patch acting as an ectopic pacemaker (Liu et al., 2018; Romagnuolo et al., 2019). Although a first attempt to transplant human ES cell-derived cardiomyocytes to a patient has been reported (Menasché et al., 2015), the arrythmias observed in animal experiments currently represent a serious concern, which will require further research to address.

## The International Society for Stem Cell Research

Early in the 21^st^ century, the technology to generate human ES cells was established, and there was a debate in several countries, not least in the US, on how the use of, and generation of, novel, ES cell lines should be regulated. These questions were also discussed among stem cell scientists, but there was no organized international forum for exchanging ideas, showcasing the latest research results, and discussing the ethical implications of stem cells and their use in future therapy development. As a response to this, Leonard Zon started the International Society for Stem Cell Research (ISSCR), with the ambition to promote the science in the field but also to engage in public outreach and communication, advocacy, and policy—a choice that may seem obvious now but was not obvious then.

**Figure 2:**
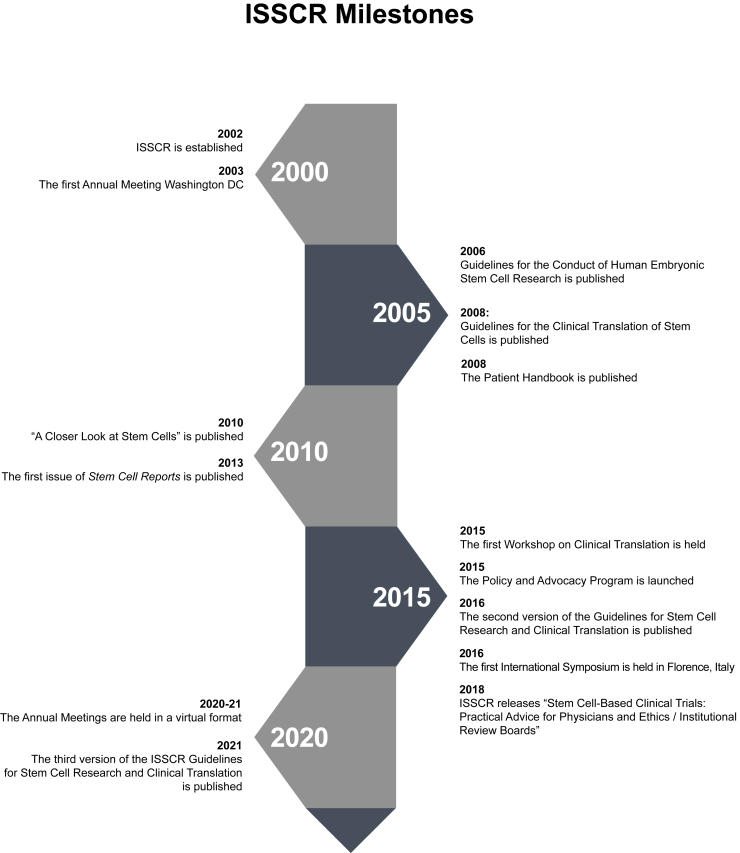
Time axis for ISSCR milestones

The overarching mission of ISSCR is the promotion of excellence in stem cell science and in translation of research for the benefit of human health. In 2003, ISSCR organized its first Annual Meeting, which since then has been an integral part of ISSCR’s activities (for a timeline of milestone events in ISSCR, see Figure 2). The Annual Meetings have grown in size from around 500 participants during the first years to 3,000–4,000 participants from over 60 countries in recent years (see Figure S1 for a list of the Annual Meetings). The majority of the Annual Meetings have been held in the US but also span the globe, and to widen its geographical footprint and to complement the large-scale Annual Meeting with a more intimate meeting format, ISSCR launched so-called International Symposia (see Figure S1 for a list of all International Symposia). The first International Symposium was held in Shanghai in 2008, and over the years 18 physical and two virtual International Symposia, which accommodate 250–500 participants, have been held in nine countries. In 2015, the Workshop on Clinical Translation, as a part of the Annual Meeting, was established to broaden the interface to the translational and clinical community. Along similar lines, the “Stem Cells Clinical Trials: Practical Advice for Physicians and Ethics/Institutional Review Boards” was published in 2018.

In 2006, ISSCR took a bold step by publishing a first set of Guidelines for Stem Cell Research and Clinical Translation (hereafter called the Guidelines). Recognizing the need to lay out principles for how stem cell research should be conducted in an ethically sound way and with high research integrity, ISSCR published the first set of Guidelines in 2006. New versions of the Guidelines appeared in 2008, 2016, and 2021, and the focus of each version reflects where the field of stem cell research stood at the time and what technologies were emerging. The 2006 edition of the Guidelines thus placed a strong focus on human ES cells, whereas later versions have incorporated recommendations, for example, for iPS cell, organoid, blastoid, editing of the human genome, and embryo research and for interspecies chimera research. The Guidelines have been proactive in providing recommendations for emerging fields, for example by including recommendations for mitochondrial replacement techniques (MRT), a technology currently allowed only in the UK, in the 2021 edition. By providing principled recommendations for how research and its application should be conducted, and whether some of the currently widely accepted regulatory frameworks should be updated or altered, the Guidelines has, inevitably in some cases, stirred a debate in the stem cell community. One such example is the suggestion to modify the so-called 14-day limit for research on human embryos, which dates back to the 1980s (McLaren, 1984) and is enshrined in law in more than 10 countries (Cavaliere, 2017). The notion that human embryos could be sustained *in vitro* for up to 13 days after fertilization (Deglincerti et al., 2016; Shahbazi et al., 2016) triggered an interest in revisiting the 14-day limit (Hyun et al., 2021; McCully, 2021). The 2021 Guidelines calls for a broader discussion on extending the time limit for embryos in culture beyond 14 days under special circumstances and with appropriate oversight (Lovell-Badge et al., 2021; Master et al., 2021). The call to open discussion on the 14-day rule has been challenged by some scientists (Green et al., 2021; Johnston et al., 2021). Another area of discussion regards ISSCR’s position on editing of the human germ line. Genomic editing for the germ line is currently prohibited in most countries, and the 2021 Guidelines agrees that clinical application should be prohibited at this time but recommends that research be supported. This has led to a debate, as some hold the view that the germ line should be sacrosanct and spared from genome-editing exercises altogether (Baylis, 2021).

As stem cell research and the marketing of unproven stem cell therapies entered the public’s awareness, ISSCR developed information about stem cell research directly for the general public. In 2008, ISSCR published the “Patient Handbook,” which in 12 languages provides answers to frequently asked questions about stem cell therapy and has since been updated. Two years later, in 2010, another public education initiative was launched, when the “A Closer Look at Stem Cells” Website was introduced, an award-winning initiative to provide easy-to-grasp information about many aspects of stem cell biology and its impact on human health, including fact-based information on stem cell therapies and so-called unproven (a.k.a. “snake oil”) stem cell-based therapies. ISSCR has been very active in terms of advocacy, notably by launching a policy and advocacy program in 2015, and by informing about the risks of unproven therapies. ISSCR members have on several occasions testified on behalf of ISSCR before governing bodies around the globe to support and defend important scientific principles and have provided expert testimony on issues such as ES cell and human fetal research, MRT, and unproven therapies.

In 2013, ISSCR took another major step, when the first issue of the journal *Stem Cell Reports* was published. *Stem Cell Reports*, which is published by Cell Press, Elsevier, has established itself as a leading journal in the field, currently publishing more than 200 articles per year and with a journal impact factor of 7.7 (2020). During its first 20 years, ISSCR has furthermore established several awards to recognize important scientific discoveries or other outstanding achievements by stem cell scientists (for a complete list of the ISSCR awards and awardees, see Figure S2). The Anne McLaren Memorial Award was established in 2008, followed by the Outstanding Young Investigator Award in 2009 (since 2018 called the Dr. Susan Lim Award for Outstanding Young Investigator), and the McEwen Award for Innovation (since 2018 called the ISSCR Award for Innovation) the Public Service Award and the Ernest McCulloch Memorial Lecture were established in 2010. In 2016, the Tobias Award Lecture was established, followed by the ISSCR Achievement Award and the Momentum Award in 2020 Figure S2.

ISSCR, like many other societies, was hit by the COVID-19 pandemic, and the 2020 and 2021 Annual Meetings needed to be switched from a physical to a digital meeting format. ISSCR, however, rapidly managed to gain the necessary expertise in arranging virtual meetings. The newly learned skills in digital content creation and meeting organization will also be useful going forward into the post-pandemic era, where a mix of real-life, hybrid, and virtual meetings will likely be the norm for ISSCR and many other societies. The ability to create professional virtual content is also increasingly used by ISSCR to produce courses, workshops, and activities such as ISSCR Digital. This contributes to a more dynamic, “year-round active” society, an improvement over the traditionally strong focus on the Annual Meeting with relatively few other activities spaced out throughout the rest of the year.

## Concluding remarks

During its first 20 years, ISSCR has made remarkable progress and established itself as a trusted voice for stem cell research, ethics, policy, and advocacy, and is providing an expanding portfolio of scientific meetings. ISSCR has also helped to call out unproven cell therapies and importantly published guidelines for how to conduct stem cell research with scientific and ethical integrity. With the current pace of progress in stem cell research, it will be interesting to see what new topics will be presented at future ISSCR meetings and addressed in new editions of the Guidelines. I, however, rest assured that ISSCR will be able to handle these tasks in a scholarly and wise manner. I wish ISSCR the best of luck in these future endeavors and a happy 20^th^ birthday!

## Conflicts of interest

U.L. holds a research grant from Merck KGaA but no personal remuneration. U.L. is a member of the Editorial Board of *Stem Cell Reports*.

## Supplemental information

Document S1. Figures S1 and S2

Document S2. Article plus supplemental information
