# Supplementary material for: 100 plus years of stem cell research—20 years of ISSCR
Source: Stem Cell Reports. 2022 Jun 14;17(6):1248–67. doi: 10.1016/j.stemcr.2022.04.004 (PMC9213821; doi:10.1016/j.stemcr.2022.04.004)
Supplement: Document S1. Figures S1 and S2 [file mmc1.pdf]

Stem Cell Reports, Volume *17*

Supplemental Information

100 plus years of stem cell research—20 years of ISSCR

Urban Lendahl

ISSCR Annual Meetings and International Symposia

Annual Meetings:
2021 – virtual meeting because of the COVID-19 pandemic (originally planned to be held in Hamburg, Germany)
2020 – virtual meeting because of the COVD-19 pandemic (originally planned to be held in Boston, US)
2019 – Los Angeles, US
2018 – Melbourne, Australia
2017 – Boston, US
2016 – San Francisco, US
2015 – Stockholm, Sweden
2014 – Vancouver, Canada
2013 – Boston, US
2012 – Yokohama, Japan
2011 – Toronto, Canada
2010 – San Francisco, US
2009 – Barcelona, Spain
2008 – Philadelphia, US
2007 – Cairns, Australia
2006 – Toronto, Canada
2005 – San Francisco, US
2004 – Boston, US
2003 – Washington DC, US

International Symposia:
2021 – virtual meetings because of the COVID-19 pandemic
2020 – virtual meetings because of the COVID-19 pandemic
2019 – Amsterdam, the Netherlands
Seoul, South Korea
Toronto, Canada
2018 - Lausanne, Switzerland
2017 - Basel, Switzerland
Guangzhou, China
2016 - Dresden, Germany
Florence, Italy
Kyoto, Japan
2015 - Sozhou, China
2014 - San Diego, US
Singapore
2013 - Florence, Italy
Sozhou, China
2012 - Boston, US

San Francisco, US
Sao Paolo, Brazil
2011 - Sozhou, China
2010 - Amsterdam, the Netherlands
Boston, US
2009 - Buenos Aires, Argentina
2008 - Shanghai, China

Supplementary Figure 1:
A list of ISSCR Annual Meetings and International Symposia.

ISSCR Awards and Awardees

ISSCR Dr. Susan Lim Award for Outstanding Young Investigator (2009-2017 called the ISSCR Outstanding Young Investigator Award)

The ISSCR Dr. Susan Lim Award for Outstanding Young Investigator, supported by the Dr. Susan Lim Endowment for Education and Research Ltd., recognizes the exceptional achievements of an investigator in the early part of their independent career in stem cell research.

Awardees:
2021 - Madeline Lancaster
2020 - Allon M. Klein
2019 – Barbara Treutlein
2018 – Shuibing Chen
2017 – Jayaraj Rajagopal
2016 – Fernando Camargo
2015 – Paul Tesar
2014 – Valentina Greco
2013 – Marius Wernig
2012 – Cédric Blanpain
2011 – Robert Blelloch
2010 – Joanna Wysocka
2009 – Konrad Hochedlinger

ISSCR Achievement Award (introduced in 2020)

The ISSCR Achievement Award recognizes the transformative body of work of an investigator that has had a major impact on the field of stem cell research or regenerative medicine.

Awardees:
2021 – Janet Rossant

ISSCR Momentum Award (introduced in 2020)

The ISSCR Momentum Award recognizes the exceptional achievements of an investigator whose innovative research has established a major area of stem cell-related research with a strong trajectory for future success.

Awardees:
2021 – Valentina Greco

ISSCR Public Service Award (introduced in 2011)

The ISSCR Public Service Award is given in recognition of outstanding contributions of public service to the fields of stem cell research and regenerative medicine.

Awardees:
2021 – Robin Lovell-Badge
2020 – Susan L. Solomon
2019 – Eli and Edythe Broad
2018 – Megan Munsie
2017 – George Q. Daley
2015 – Alan Trounson
2014 – Paolo Bianco, Elena Cattaneo, Michele De Luca
2013 – Hiromitsu Ogawa, Betty Jean Crouch Ogawa
2012 – Rob and Cheryl McEwen
2011 – Robert Klein

ISSCR Tobias Award Lecture (2016-2021)

The ISSCR Tobias Award Lecture was established in 2015 by the Tobias Foundation. The award recognized original and promising basic hematology research as well as direct translational or clinical research related to cell therapy in hematological disorders.

Awardees:
2021 – Stuart H. Orkin
2020 – Margaret A. Goodell
2019 – Scott Armstrong
2018 – Connie Eaves
2017 – John Dick
2016 – Leonard Zon

ISSCR Award for Innovation (2018-2019; 2011-2017 called the McEwen Award for Innovation)

The ISSCR Award for Innovation recognized a transformative breakthrough that has had a major impact on the field of stem cell research or regenerative medicine.

Awardees:
2019 - John Dick
2018 – Michele De Luca, Graziella Pellegrini
2017 – Elaine Fuchs
2016 – Austin Smith, Qi-Long Ying
2015 – Hans Clevers, Irving Weissman
2014 – Azim Surani
2013 – James A. Thomson
2012 – Rudolf Jaenisch
2011 – Kazutoshi Takahaski, Shinya Yamanaka

Other awards:

The ISSCR Zhongmei Chen Yong Awards for Scientific Excellence (travel awards)
ISSCR Poster Awards
ISSCR Merit Abstract Awards

Supplementary Figure 2:
A list of ISSCR Awards and Awardees.
