# Supplementary material for: 100 plus years of stem cell research—20 years of ISSCR
Source: Stem Cell Reports. 2022 Jun 14;17(6):1248–67. doi: 10.1016/j.stemcr.2022.04.004 (PMC9213821; doi:10.1016/j.stemcr.2022.04.004)
Supplement: Document S2. Article plus supplemental information [file mmc2.pdf]

Stem Cell Reports
Perspective
ISSCR
OPEN ACCESS

100 plus years of stem cell research—20 years of ISSCR

Urban Lendahl[1,*]
[1]Department of Cell and Molecular Biology, Karolinska Institutet, 17177 Stockholm, Sweden
*Correspondence: urban.lendahl@ki.se
https://doi.org/10.1016/j.stemcr.2022.04.004

SUMMARY

The International Society for Stem Cell Research (ISSCR) celebrates its 20th anniversary in 2022. This review looks back at some of the key developments in stem cell research as well as the evolution of the ISSCR as part of that field. Important discoveries from stem cell research are described, and how the improved understanding of basic stem cell biology translates into new clinical therapies and insights into disease mechanisms is discussed. Finally, the birth and growth of ISSCR into a leading stem cell society and a respected voice for ethics, advocacy, education and policy in stem cell research are described.

STEM CELL RESEARCH—THE EARLY YEARS

Stem cells are defined by the ability to self-renew and to produce differentiated cells, but what could be considered the starting point of this research field? A defining moment is difficult to identify precisely, but an important conceptual prerequisite for stem cell research, and in fact for all cell biology, was the development of the cell theory in the mid-1800s by Rudolf Virchow, Rudolf Remak, and Theodor Schwann and the realization that all cells are derived from other cells through cell division – "*omnis cellula a cellula*" (Virchow, 1858). The first descriptions of the word stem cell also date back to the mid-1800s. Ernst Haeckel used the term "*Stammzellen*" in 1868, but originally in a more phylogenetic context, to denote a unicellular organism from which multicellular organisms developed. In 1877 he extended its use to the fertilized egg, in line with his concept of "ontogeny recapitulates phylogeny" (Haeckel, 1877) (see Figure 1 for a timeline of some of the key discoveries in stem cell research). Theodor Heinrich Boveri and Valentin Häcker used stem cell as a term for cells giving rise to the germ line, thus expanding its use to cell types other than the fertilized egg cell. Häcker also made the important observation that cell division in the crustacean *Cyclops* led to one cell remaining as a stem cell while the other cell differentiated (Haecker, 1892)—an early observation of asymmetric cell division. Boveri characterized cells giving rise to germ cells and somatic cells and referred to them as stem cells (for review see Maehle, 2011). After the initial use of stem cells referring to the germ line, Alexander Maximow, Wera Dantschakoff, and Artur Pappenheim started using the term stem cell in the context of hematopoiesis to denote cells producing the different types of cells in the blood (Dantschakoff, 1908; Maximow, 1909; Pappenheim, 1896).

STEM CELL RESEARCH MILESTONES—LESSONS FROM FOUR ORGAN SYSTEMS

Progress in stem cell research was reported from many frontiers during the 1900s. In the pre-molecular era, transplantation experiments in amphibians yielded new insights into the role of communication between various organs and tissues for cellular differentiation (Spemann and Mangold, 1924). The development of transgenic technologies, more advanced cell culturing techniques and decoding of genomes from various species further fueled progress in our understanding of stem cells and their various differentiation trajectories. A detailed account of this era is beyond the scope of this review, and I will instead focus on discussing some of the landmark discoveries in stem cell research in four mammalian organs—the hematopoietic system, the skin, the CNS, and the intestine—as each of these organs has provided important concepts for the field at large and revealed opportunities for clinical translation and understanding of disease processes.

The hematopoietic system

As discussed above, Pappenheim, Maximow, and Dantschkoff introduced the stem cell concept for hematopoiesis, and Pappenheim, in fact, produced a scheme for a hematopoietic stem cell hierarchy not too distant from the version that is agreed upon today (Ramalho-Santos and Willenbring, 2007). It was, however, extensively discussed whether there was a single or more than one type of stem cells for the blood, and there were two camps, dualists and unitarians, with different views on this subject. The unitarian Ernst Neumann observed that hematopoiesis takes place in the bone marrow and suggested that one cell can give rise to all the different blood cells (Neumann, 1868), whereas Paul Ehrlich, for example, advocated separate origins for the different types of cells in the blood (Ramalho-Santos and Willenbring, 2007).

This issue took considerable time to resolve, as hematopoietic stem cells amount to less than 0.01% of all bone marrow cells. An initial landmark discovery in hematopoietic research, and for stem cell research in general, was the first functional assay to quantitate hematopoietic stem

This is an open access article under the CC BY license (http://creativecommons.org/licenses/by/4.0/).

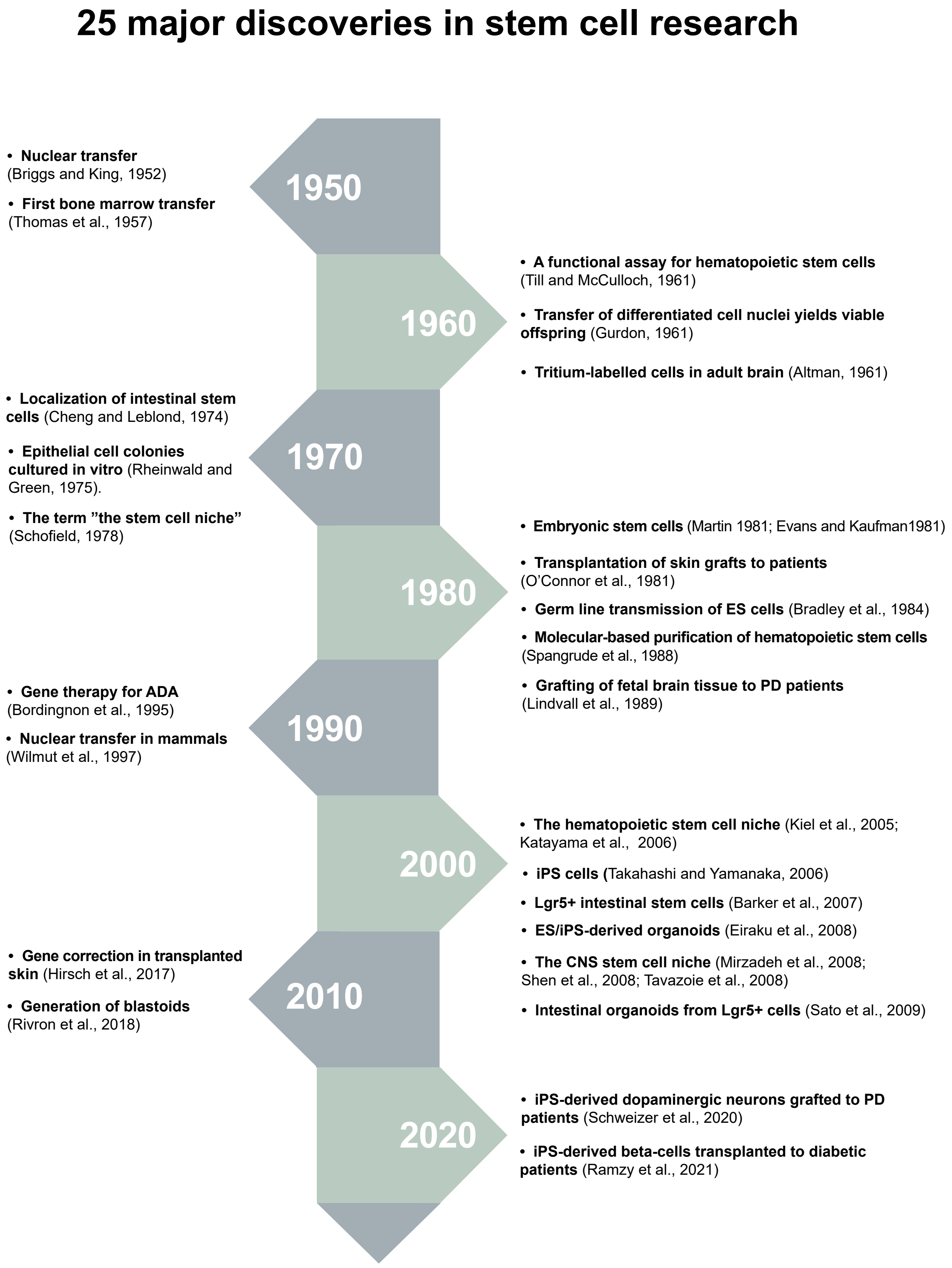

Figure 1. Time axis for 25 major discoveries in stem cell research

cells (or at least closely related multipotent progenitors). This was accomplished by James Till and Ernest McCulloch, who, on the basis of the discovery that bone marrow cells could be successfully transplanted to an irradiated mouse host (Ford et al., 1956), demonstrated that hematopoietic stem cells were endowed with self-renewing as well as multi-lineage differentiation capacities (Becker et al., 1963; Till and McCulloch, 1961). Irving Weissman's laboratory, building on the Till and McCulloch discoveries, combined the recently invented fluorescence-activated cell sorting (FACS) technology with the use of novel monoclonal antibodies and a negative selection strategy, to provide the first set of molecular markers (Thy1.1^{lo}-Sca-1^{hi}-Lin$^{-/lo}$) that gave high enrichment of hematopoietic stem cells. As few as 30 such cells were sufficient for 50% survival in transplanted lethally irradiated mice and reconstituted all hematopoietic cell types (Spangrude et al., 1988). Several observations, however, suggested that the Thy1.1^{lo}-Sca-1^{hi}-Lin$^{-/lo}$ cells remained heterogeneous and that further subdivision of the cells resolved them into long-term self-renewing, short-term self-renewing, and non-self-renewing multipotent progenitors (Morrison and Weissman, 1994). Subsequent work further refined the molecular definition of hematopoietic stem cells (Kiel et al., 2005), but several lines of evidence, including analysis of their epigenetic state and dormancy, suggested that the hematopoietic stem cell pool is molecularly heterogeneous (Foudi et al., 2009; Oguro et al., 2013; Wilson et al., 2008; Yu et al., 2016).

Based on the success in transplanting bone marrow in mice (Ford et al., 1956), it was realized early on that transplantation of hematopoietic stem cells could have huge medical potential, providing an opportunity to replace an ailing or cancerous human hematopoietic system. In 1957, Donnell Thomas and colleagues performed the first allogeneic (from a genetically different individual) bone marrow transplantation in humans (Thomas et al., 1957). Although the first six patients died within 100 days, there were indications of a "take" of the donor bone marrow cells, demonstrating that the concept as such was viable. Georges Mathé a few years later realized that the immunological reaction of the grafted cells toward the cells in the recipient host could be harnessed as a means to help rid the body of remaining cancerous cells, and this controlled graft-versus-host reaction saved patients with relapsing lymphoblastic leukemia that had been transplanted by mixed-donor bone marrow cells (Mathé et al., 1965). The range of diseases that could be treated was gradually extended, and allogeneic transfer was used to replace the hematopoietic system in patients suffering from severe combined immunodeficiency (SCID) and Wiskott-Aldrich syndrome (Bach et al., 1968; Gatti et al., 1968). Following these early pioneering discoveries, further progress in what is now referred to as allogeneic hematopoietic stem cell transfer (allo-HSCT) has been made with regard to immunological matching and immunosuppression, and allo-HSCT is today used routinely in the clinic. To improve the donor base, efforts have been made to establish international registries of unrelated donors, such as the World Marrow Donor Association (Lown et al., 2014). Furthermore, the repertoire of donor cell sources has been expanded to include, for example, umbilical cord blood cells, which, however, take longer to reconstitute the hematopoietic system (see Cieri et al., 2021 for review).

The CNS

The brain and spinal cord constitute the CNS and arise from stem cells in the embryonic neuroectoderm. Induction of neuroectoderm by the underlying mesoderm was initially demonstrated by Hilde Mangold and Hans Spemann through transplantation experiments in amphibians (Spemann and Mangold, 1924). Retroviral lineage-tracing experiments revealed the existence of embryonic neural stem cells giving rise to both neurons and glial cells (Price and Thurlow, 1988; Turner and Cepko, 1987), and a similar lineage bipotentiality was observed for cultured neural stem cells (Cattaneo and McKay, 1990; Davis and Temple, 1994). A few years later, it was demonstrated that radial glial cells, previously assumed to have more structural roles, served as neural stem cells in the embryonic brain (Malatesta et al., 2000; Noctor et al., 2001). How neural stem cells proceed to acquire specific neuronal identities and the role of transcription factors and morphogens in this process was elucidated by the late Tom Jessell and colleagues (Briscoe et al., 1999; Liem et al., 1997).

The question whether stem cells persisted in the adult brain was a thornier question and a matter of considerable debate for many years. Wilhelm His, in fact, observed cells with mitotic figures near the ventricles of the adult human brain almost 150 years ago (Breunig et al., 2011; His, 1874), supporting that cell division indeed took place. This notion was, however, disputed by many leading contemporary neurobiologists, including Ramón y Cajal, who remained skeptical and argued that "nothing changed after development" (Breunig et al., 2011; Takagi, 2016). In the 1960s, the technique to label dividing cells with tritiated thymidine *in vivo* was developed (Smart and Leblond, 1961). Joseph Altman used this technique to identify cell divisions in the adult rat subventricular zone (SVZ) and dentate gyrus and to show that dividing cells born in the postnatal SVZ migrated along a rostral migratory stream to the olfactory region (Altman, 1961, 1969; Altman and Das, 1965). Adult neurogenesis was not confined only to mammals but was also reported in canary birds, in which new neurons are generated yearly in association with song behavior

(Goldman and Nottebohm, 1983). An indication that adult neurogenesis occurs in humans was provided by Fred Gage and the late Peter Eriksson, who identified label-retaining cells in *post mortem* brains from patients who had received bromodeoxyuridine (BrdU) for diagnostic purposes in conjunction with tumor therapy (Eriksson et al., 1998). The precise location of the adult CNS stem cells near the ventricles of the brain was for some time a matter of debate (Doetsch et al., 1999; Johansson et al., 1999), but the current model holds that stem cells with primary cilia (type B cells) give rise to transient-amplifying cells (type C cells), which subsequently become neuroblasts (type A cells). Adult CNS stem cells are largely quiescent (Doetsch et al., 1999), but a certain amount of cellular turnover in the human brain has been demonstrated by Jonas Frisén and colleagues, who developed a unique technique to birth-date cells based on their cellular carbon-14 content, taking advantage of the "spike" in atmospheric carbon-14 levels resulting from above-ground nuclear testing in the early 1960s (Spalding et al., 2005, 2013). The extent to which there is adult neurogenesis in specific human brain regions, such as the dentate gyrus of the hippocampus, is, however, still intensely debated, and reports arguing for (Boldrini et al., 2018; Spalding et al., 2013) or against (Franjic et al., 2022; Sorrells et al., 2018) adult hippocampal neurogenesis have been presented (for review see Kempermann et al., 2018; Paredes et al., 2018).

Skin

The history of stem cells in the skin is strongly centered around one scientist, Howard Green, who not only pioneered skin transplantation in the clinic but also worked out important cellular principles and trained a cadre of today's leading epithelial biology scientists. Howard Green started his research in the early 1970s by studying teratomas (as many other scientists did at the time; see below) and in these studies he noted that epithelial cells formed colonies in cell culture and that their ability to expand was enhanced by culturing them on feeder cells (Rheinwald and Green, 1975). Next, he developed procedures for detaching the cultured epithelial sheets (Green et al., 1979) and succeeded in transplanting them to mice (Banks-Schlegel and Green, 1980). In a landmark study, Green transplanted two patients with third-degree burn injuries with autologous skin grafts (O'Connor et al., 1981), which represented the first step toward a life-saving therapy for patients with severe burn injuries. Skin transplantation is nowadays well established in the clinic and has more recently been combined with gene correction techniques: a patient with junctional epidermolysis bullosa was grafted with skin in which the *LAMB3* gene was inserted to provide correct expression of laminin-332 (Hirsch et al., 2017).

Skin constitutes approximately 15% of body weight and is composed of two layers (epidermis and dermis), as well as hair follicles, sweat glands, and sebaceous glands. Epidermal stem cells are located in the basal layer (Barrandon and Green, 1987) and balance self-renewal and production of keratinocytes that progress through the upper, suprabasal layers, eventually ending up as dead squames, which are shed from the stratum corneum (for review see Blanpain and Fuchs, 2009). Differentiation is accompanied by a coordinated change in keratin expression to appropriately adapt the cytoskeleton of the keratinocytes to their position in the epidermis (Fuchs and Green, 1978, 1980). The choice between self-renewal and differentiation of the epidermal stem cells is, at least in part, controlled by asymmetric cell division and the angle of the cleavage plane (Lechler and Fuchs, 2005; Smart, 1970). Regulation of clone size and distribution of clones derived from individual epidermal stem cells and whether clone expansion involves an intermediate transient-amplifying cell population are topics that have been intensely studied in the mouse. Models based on stochastic events, neutral drift, or distinct stem cell pools and lineages have been proposed (Clayton et al., 2007; Gomez et al., 2013; Jones and Watt, 1993; Mascré et al., 2012; for review see Rognoni and Watt, 2018).

In addition to the stem cells residing in the basal layer, the hair follicles contain molecularly distinct stem cell populations positioned at different locations of the hair follicle, including the bulge region and the sebaceous gland (Cotsarelis et al., 1990; Jensen et al., 2009; Tumbar et al., 2004). While bulge stem cells normally give rise only to the hair follicle, lineage tracing and transplantation experiments revealed that they can contribute to both hair and epidermis (Blanpain et al., 2004; Claudinot et al., 2005). A specific feature of the hair follicle stem cells is that they need to tune their activity to the hair cycle, which switches between a resting (telogen), a regenerative (anagen), and a destructive (catagen) phase (Blanpain and Fuchs, 2009). Fgf18 and BMP6 produced from differentiating cell progeny play key roles for maintaining quiescence (Hsu et al., 2011), while SHH produced from transient-amplifying cells activates the stem cells (Hsu et al., 2014). In the normal, non-injured state, the various stem cell populations give rise to distinct subsets of differentiated cells (Jensen et al., 2009; Page et al., 2013), but in response to injury, all stem cell populations give rise to epidermal cells (Aragona et al., 2017; Donati et al., 2017; Ge et al., 2017; Park et al., 2017). This "all hands on deck" stem cell contribution to epidermal cells upon injury is likely important to rapidly heal an epidermal wound. Interestingly, the response of epidermal stem cells may be tuned by previous experiences, such as acute inflammation (Naik et al., 2017).

Intestine

The intestine is a hostile environment for cells, and in line with this, there is a very rapid cell turnover, with an average life span of only a few days for intestinal cells. The intestine is composed of two principal categories of cells: absorptive (enterocytes and M cells) and secretory (goblet, Paneth, tuft, and enteroendocrine cells) cells, and both categories originate from intestinal stem cells (Beumer and Clevers, 2021). The tiny crypt-base columnar (CBC) cells, localized at the bottom of the intestinal crypts between the much larger Paneth cells, were first proposed as the enigmatic crypt stem cell (Cheng and Leblond, 1974). Later, Chris Potten proposed that cells located at position +4 (counting from the crypt base) rather than the CBC cells constituted the "real" stem cell (Potten et al., 2002), which remained the dominant view for years. In an early version of genetic lineage tracing, cell progeny born at or near the crypt base were demonstrated to migrate away from the crypts along the crypt-villus axis (Winton et al., 1988). The discovery that the crypt-base columnar cells express Lgr5 provided definitive, lineage-tracing-based evidence in support of CBCs as the crypt stem cell (Barker et al., 2007). An interesting feature of the Lgr5$^+$ intestinal stem cells is that they constantly divide and show high telomerase activity (Sato et al., 2009; Schepers et al., 2011), which sets them apart from quiescent stem cells in many other organs. Lineage tracing of the intestinal stem cells by virtue of their Lgr5 expression has provided insights into clonal distribution of differentiated cell progeny (Barker et al., 2007), and neutral drift and competition between the stem cells eventually leads to clonality in the crypts (Lopez-Garcia et al., 2010; Snippert et al., 2010). More recently, a slow-dividing Lgr5$^+$ cell population has been identified, which normally gives rise to Paneth and enteroendocrine cells, but upon injury can generate all intestinal cell types (Buczacki et al., 2013). An intriguing finding is that more differentiated intestinal cell types can revert to become intestinal stem cells upon injury (van Es et al., 2012; Jadhav et al., 2017; Tetteh et al., 2016), thus helping to replenish the intestinal stem cell pool.

THE STEM CELL NICHE

Stem cells do not function in splendid isolation; they are, in fact, highly dependent on interactions with surrounding cells and tissues, which constitute the stem cell niche. Ray Schofield launched the concept of a stem cell "niche" in the hematopoietic system (Schofield, 1978), and considerable progress has since then been made in terms of characterizing the hematopoietic stem cell niche. In the 1970s, Michael Dexter and colleagues showed that stromal cells were important for culturing of hematopoietic stem cells *in vitro* (Dexter et al., 1977). Next, osteoblasts in the bone marrow were considered to be the important niche cells (Calvi et al., 2003; Zhang et al., 2003), but results from subsequent studies have instead revealed that hematopoietic stem cells reside near sinusoidal blood vessels in the bone marrow (Kiel et al., 2005, 2007; Sugiyama et al., 2006), suggesting that the endothelial or paravascular cells provide the important niche signals and that the osteoblasts exert more indirect effects. Indeed, leptin receptor-positive stromal cells, together with endothelial cells, produce factors such as stem cell factor (SCF) and Cxcl12, which are critical for stem cell maintenance (Ding and Morrison, 2013; Ding et al., 2012; for review see Comazzetto et al., 2021; Morrison and Scadden, 2014). Furthermore, the late Paul Frenette demonstrated that the sympathetic nervous systems provided signals for hematopoietic stem cell mobilization (Katayama et al., 2006). Hematopoietic stem cell maintenance is also influenced by various types of immune cells, such as granulocytes and monocytes, located at specific sites in the bone marrow (Hérault et al., 2017; Zhang et al., 2021), as well as by stress conditions (Severe et al., 2019), offering mechanisms by which changes in overall physiological status can be sensed and influence hematopoietic stem cell activity.

In the adult brain, stem cells are primarily located in the subventricular zone and the hippocampus, and progress has been made in decoding their niches. In the subventricular zone, the stem cells reside in pinwheel-like niche structures near the brain vasculature and cerebrospinal fluid (Mirzadeh et al., 2008; Shen et al., 2008; Tavazoie et al., 2008), while neural stem cells in the hippocampus are positioned close to the inner granule cell layer in the dentate gyrus (Sun et al., 2015). How changes in niche composition, for example with regard to nutrient sensing, contributes to the cognitive decline observed during aging is an emerging research area (for review see Navarro Negredo et al., 2020). Other, somewhat less intuitive, and longer-range niche components in the brain are the meninges, which are membranous structures circumscribing the brain, and the choroid plexus, the major source of cerebrospinal fluid production. Both the meninges and the choroid plexus produce factors that influence neural stem cells, such as CCL2, CXCL12, and retinoic acid (Belmadani et al., 2015; Radakovits et al., 2009; Siegenthaler et al., 2009; Silva-Vargas et al., 2016). The microenvironment for oligodendrocyte progenitor cells has been shown to stiffen with age in the brain, which contributes to an age-related decline in oligodendrocyte production (Segel et al., 2019).

In the skin, the epidermal stem cells in fact contribute to shaping their own niche by producing the extracellular matrix on which they sit in the basal layer (Blanpain and

Fuchs, 2009). The extracellular matrix also provides a reference point for the stem cell division plane, dictating the balance between self-renewal and differentiation (see above). External stimuli can affect the niche to regulate epidermal stem cell activity, and such stimuli include mechanical stretching of the skin (Aragona et al., 2020; Fiore et al., 2020), as well as stress and activity in the sympathetic innervation to the skin (Shwartz et al., 2020; Zhang et al., 2020). Furthermore, alterations in the epidermal stem cell secretome modulate surrounding lymphatic capillaries, which constitute part of the epidermal stem cell niche (Gur-Cohen et al., 2019). For hair follicle stem cells, the dermal papilla, located adjacent to the bottom of the hair follicle, is a driver of stem cell activation, and immune cells (T cells and macrophages) also influence hair follicle stem cells (Ali et al., 2017; Castellana et al., 2014).

Paneth cells, i.e., the cells intercalated between the $Lgr5^+$ stem cells, constitute an important niche component for intestinal stem cells (Sato et al., 2011). If Paneth cells are experimentally ablated, they can be replaced by other cells, such as enteroendocrine cells, that take over the niche function (van Es et al., 2019; for review see McCarthy et al., 2020). Paneth cell replacement, along with replacement of ailing or lost intestinal stem cells by more differentiated cells (see above), provides important mechanisms for safeguarding intestinal cell turnover and contributes to making the intestine quite resilient to injury. More “long-range” niche signals provided to the intestinal stem cells have also been identified, with mesenchymal cells (a.k.a. myoepithelial cells or telocytes) in the vicinity of the crypts and villi providing secreted factors such as Wnt, R-spondin, and BMP-inhibitors (Beumer and Clevers, 2021). Analysis of stem cell niches in different organs is a very active research field, and further progress is expected regarding the response of the niches to altered physiological conditions, injury, and age, and to shed light on how stem cells themselves contribute to the niche (Fuchs and Blau, 2020; Gola and Fuchs, 2021).

THE QUEST FOR CELLULAR REJUVENATION AND PLURIPOTENCY

In addition to understanding the underpinning mechanisms of stem cell maintenance and differentiation, there was a parallel interest in learning whether the phenotype of a differentiated cell could in some way be reversed, leading to “rejuvenation” of a mature cell. This was first explored by asking whether a differentiated cell nucleus could revert to a more immature state if transferred to an enucleated, undifferentiated cell. The idea of nuclear transfer was already contemplated by Hans Spemann, but it was Robert Briggs and Thomas King who showed that such an experiment was technically possible, by demonstrating that nuclei from frog blastula transplanted into enucleated frog eggs gave rise to tadpoles (Briggs and King, 1952). John Gurdon, using the Briggs and King somatic cell nuclear transfer (SCNT) technology, then provided the first demonstration that tadpoles could be produced after transplantation of a cell nucleus from a differentiated adult frog cell (Gurdon, 1962). Following this pioneering report, SCNT was established in mammalian species, including sheep (Wilmut et al., 1997) and mice, where a combination of SCNT and gene therapy could correct a genetic defect in the nuclear donor strain (Rideout et al., 2002). SCNT has also found novel medical uses, for example in the mitochondrial replacement technique (MRT), an emerging strategy to correct mitochondrial disease in humans. MRT rests on a combination of *in vitro* fertilization techniques originally pioneered by Patrick Steptoe and Robert Edwards (Steptoe et al., 1971) and SNCT and is used as a means for mothers carrying severe mtDNA mutations to have genetically related children (Cohen et al., 2020). Technically, by transferring the male and female pronuclei from a fertilized egg from parents with mitochondrial disease by pronuclear DNA transfer (PNT) into an enucleated donor zygote, or alternatively transferring the metaphase II spindle complex from the mother’s oocyte into an enucleated donor oocyte, the faulty mitochondria from the mother are replaced with those from the donor zygote or oocyte (see also below under the ISSCR guidelines).

To learn to culture the most undifferentiated cells *in vitro* represented another Holy Grail for stem cell research, as it was expected to give insights into cellular pluripotency and how such a state could be maintained. It was argued that pluripotent cells should reside in the inner cell mass of the blastocyst but possibly also in teratomas and teratocarcinomas, tumors containing a bewildering variety of differentiated cell types and tissue, suggesting the existence of highly undifferentiated stem cells in these tumors (for review see Solter, 2006). Leroy Stevens and Clarence Cook Little showed that the propensity for developing testicular teratomas, which normally is very low in mice, was elevated in a specific mouse strain, the 129-strain (Stevens and Little, 1954). This opened new vistas for gaining insights into this tumor type, and teratocarcinomas from the 129-strain could be propagated in the abdominal cavity of mice (Kleinsmith and Pierce, 1964). Subcutaneous transplantation of single cells from the ascites fluid contributed to a variety of tissues (Kleinsmith and Pierce, 1964), revealing that multipotent cells (referred to as embryonal carcinoma cells) could be identified experimentally. Ralph Brinster advanced the transplantation paradigm further by demonstrating that transfer of embryonal carcinomas cells into the early mouse blastocyst resulted in chimeric offspring (Brinster, 1974).

The question, however, remained whether cells that could give rise to all cell types in an animal, i.e., totally pluripotent cells, could be identified and harnessed *in vitro*. Gail Martin and Martin Evans managed to culture inner-cell mass-derived cells (referred to as embryonic stem [ES] cells) and produce teratocarcinomas upon transplantation (Evans and Kaufman, 1981; Martin, 1981). Three years later, Evans and colleagues demonstrated that ES cells could yield germline chimeric mice (Bradley et al., 1984). ES cell lines were next generated from non-human primates (Thomson et al., 1995) and humans (Shamblott et al., 1998; Thomson et al., 1998).

The SCNT experiments discussed above demonstrated that a differentiated cell nucleus could be rejuvenated if placed in an appropriate juvenile cellular environment; but would it be possible to revert an intact differentiated cell into an undifferentiated, pluripotent state? For some time, this remained more of a thought experiment, and the route to inducing pluripotency in a cell was viewed to be very complex, if not impossible. The molecular decoding of ES cells, however, provided insights into factors required to maintain the pluripotent state in the culture dish (Chambers et al., 2003; Nichols et al., 1998). This information was used to test combinations of such factors, and the discovery that expression of only a very small set of transcriptional regulators (Oct4, Sox2, cMyc, and Klf4) was sufficient to convert a differentiated mouse fibroblast into an induced pluripotent stem cell (iPS cell) came as a surprise to the field (Takahashi and Yamanaka, 2006). The generation of human iPS cells was published a year later (Takahashi et al., 2007; Yu et al., 2007). It was also soon demonstrated that provision of a small set of transcriptional regulators could drive a direct conversion of one type of differentiated cell into another, without proceeding through the pluripotent state. In this way, β-cells, oligodendrocytes, and neurons were produced from other differentiated cell types by direct lineage conversion (Vierbuchen et al., 2010; Yang et al., 2013; Zhou et al., 2008; for review see Falk et al., 2021). The notion that a direct lineage conversion could be obtained by expression of specific combinations of transcription factors was also in line with a classical observation by the late Harold Weintraub that expression of MyoD was sufficient to convert fibroblasts (10T1/2 cells) into myoblasts (Lassar et al., 1986).

ORGANOIDS

Protocols were developed to steer ES and iPS cell differentiation toward specific differentiated cell fates, and when combined with the introduction of disease-specific mutations into the genome of the pluripotent cells, new light was shed on the molecular basis for monogenic diseases, such as amyotrophic lateral sclerosis, Parkinson's disease, and long QT syndrome (see Soldner and Jaenisch, 2018 for review). Most of the early protocols, however, relied on culturing the cells as a flat two-dimensional (2D) monolayer, and it made intuitive sense that three-dimensional (3D) culturing of cells would more closely recapitulate the *in vivo* situation and thus be superior to 2D culturing. There was, therefore, an interest in exploring whether ES and iPS cells could be not only differentiated but guided toward forming more complex mini-organs, called organoids, when cultured in 3D (see Lancaster and Knoblich, 2014 for review). When ES or iPS cells were allowed to proceed through an embryoid body-like state, recapitulating early embryo development, they revealed signs of self-organization into organ-like structures. Pioneering research by the late Yoshiki Sasai yielded retinal and brain organoids (Eiraku et al., 2008, 2011), and subsequent work by Jürgen Knoblich's and Sasai's research groups demonstrated that brain organoids with advanced anatomical organization could be generated and that brain disease-specific features could be mimicked in the organoids (Kadoshima et al., 2014; Lancaster et al., 2013).

Adult-tissue stem cells from various epithelial structures turned out to be an alternative cellular source for organoid generation. A breakthrough in this area was the discovery that intestinal $Lgr5^+$ stem cells (see above) gave rise to organoids with many features of the intestinal crypt (Sato et al., 2009) and that such organoids engrafted successfully when transplanted to the mouse intestine (Yui et al., 2012). Organ-specific organoids have now been generated from $Lgr5^+$ stem cells from most other organs, including liver (Huch et al., 2013). Organoids not only shed light on principles for organ generation but are increasingly used to study disease mechanisms, for example in infectious disease research, where the effects of Zika virus exposure have been studied in brain organoids (Qian et al., 2016), and various organoid systems have rapidly been adapted for COVID-19 research (see Geurts et al., 2021 for review). The effects of specific disease mutations have been explored in organoid systems, including mutations causing microcephaly (Lancaster et al., 2013), CFTR (Dekkers et al., 2013) and liver diseases such as alpha1-antitrypsin and Alagille syndrome (Huch et al., 2015). Although most examples of disease modeling in organoids still come from monogenic diseases, organoids from patients with genetically more complex, non-monogenic diseases, such as biliary atresia, have also unveiled disease-specific phenotypes (Babu et al., 2020). Finally, organoids are increasingly used to unravel the molecular basis for various types of cancer and to explore personalized-medicine approaches for cancer therapy (Kastner et al., 2021). As will be discussed later, we can envisage the generation of increasingly more complex organoid systems, and analysis of interactions between organoids and specific cell types,

such as immune cells. To grow larger organoids will at some point likely require vascularization, and blood vessel organoids have recently been generated (Wimmer et al., 2019).

An interesting recent offshoot from the organoid tree is the development of blastoids, blastocyst-like structures that have spurred a new research field referred to as synthetic embryology (Li et al., 2022). Blastoids were first produced from mouse cells, where an assembly of ES cells and trophoblast stem cells produced blastocyst-like structures (Rivron et al., 2018). More recently, human blastoids have been developed that undergo lineage specification in the order expected of blastocysts and that can attach to endometrial cells (Kagawa et al., 2022; Yanagida et al., 2021; Yu et al., 2021).

TOWARD CLINICAL TRANSLATION OF STEM CELL RESEARCH

Given the vast body of basic stem cell research, how rapid is the transition of this information to clinical applications in the different organ systems? Progress is made on many fronts, but the extent of clinical translation differs between organ systems. Allo-HSCT from bone marrow, peripheral blood, or cord blood is well established and currently used routinely in the clinic to treat hematological malignancies or congenital immunodeficiencies such as β-thalassemia, Fanconi anemia, sickle cell anemia, acute and chronic leukemia, non-Hodgkin's lymphoma, and Hodgkin's disease (Ferrari et al., 2021). There is also considerable progress in developing gene correction strategies for autologous hematopoietic stem/progenitor cells. Adenosine deaminase deficiency (ADA), an immune-deficiency disorder, was cured by adding the ADA gene into a patient's bone marrow cells and peripheral blood lymphocytes, providing long-term immune system restoration (Bordignon et al., 1995). However, the use of retroviral vectors for gene corrections in hematopoietic cells initially resulted in aberrant viral integrations leading to T cell acute lymphoblastic leukemia (Howe et al., 2008), For a long time, the risk of tumor development cast a shadow over the entire gene therapy field, but improvement in viral vectors, for example through the use of self-inactivating gammaretroviral or lentiviral vectors, have enhanced efficacy and led to safer therapies (Ferrari et al., 2021). Direct gene editing, rather than virus-based gene insertions, is an interesting avenue to explore, and successful correction of the mutated *IL2RG* gene in hematopoietic stem cells from a SCID patient has been reported (Genovese et al., 2014). CRISPR-Cas9 gene modification strategies are being developed but are not yet clinically approved (Ferrari et al., 2021).

In contrast to skin transplantation and allo-HSCT, where therapies are well established in the clinic, cell replacement therapies for brain diseases are still a work in progress. Parkinson's disease (PD) has long been an attractive candidate for stem cell therapy because a specific cell type, the A9 nigral neurons providing dopaminergic innervation to striatum, are lost in PD. Furthermore, the efficacy of dopaminergic agents such as L-DOPA and levodopa declines after a few years and can cause side effects, notably dyskinesia (involuntary movements). Important steps toward PD cell therapy included proof-of-principle for survival of dopaminergic neuronal grafts in rats (Olson and Seiger, 1973) and development of a PD-mimicking rat model where dopaminergic neurons in the nigrostriatal system were chemically depleted by 6-hydroxydopamine (6-OHDA) (Ungerstedt, 1968). It was next reported that transplantation of fetal dopaminergic grafts improved outcome in the rat 6-OHDA model (Björklund et al., 1980; Freed et al., 1981; Perlow et al., 1979). A study using adrenal medullary tissue grafted into two PD patients (Backlund et al., 1985) spurred open-label trials of human fetal ventral mesencephalic allografts (Lindvall et al., 1989), which showed some evidence of clinical success and cell survival, based on PET imaging. This was followed by a series of further open-label studies and then two double-blind NIH-funded placebo-controlled studies in the US, which gave conflicting results regarding the extent of improvement, if any, that was seen in transplanted patients. The analysis was also complicated by inclusion of patients with different disease severity, the use of differing amounts of the transplanted tissue, and different levels of immunosuppression (Barker et al., 2015). It was also noted that Lewy body formation was observed in some of the grafts (Kordower et al., 2008; Li et al., 2008), indicating that the pathology may spread from host tissue to the graft.

The finding that some PD patients having received fetal grafts showed some long-term improvements (Kefalopoulou et al., 2014) was encouraging, but the use of fetal tissue is ethically problematic, and the supply of tissue is limited. Therefore, *in vitro* differentiation of ES and iPS cells along the dopaminergic neuron trajectory has been intensely pursued as an alternative source of transplantable cells. Protocols for differentiation of dopaminergic, tyrosine hydroxylase-positive neurons were established by several groups, and the realization that dopaminergic neurons were derived from floor plate cells (Bonilla et al., 2008; Ono et al., 2007) led to considerably improved protocols and differentiation efficiency (Chambers et al., 2009; Kriks et al., 2011) as well as successful outcomes on transplantation of the resulting cells into animal models (Kikuchi et al., 2017; Kirkeby et al., 2017; see Kim et al., 2020 for review).

With the data from fetal and ES/iPS transplantations at hand, clinical translation is now pursued in different projects and consortia, including GForce-PD, an international consortium to advance and harmonize stem cell-derived

dopamine cell transplant therapies for PD (Barker et al., 2015). The first PD patients have recently been grafted with iPS-derived neurons (Schweitzer et al., 2020; Takahashi, 2020; see also Kim et al., 2020; Parmar and Björklund, 2020 for review). Transplantations have recently started or will soon start also at other centers and consortia, including a Cambridge-Lund study (STEM-PD), Kyoto University (Takahashi, 2020), and Sloan Kettering (Kim et al., 2020). It will be interesting to learn how effective stem cell-based therapies will be for PD patients across the various consortia and how such therapies will compare with other therapies for advanced PD, such as deep brain stimulation (DBS) (Barker et al., 2021).

Transplantation of different retinal cell types is an interesting avenue to restore vision for patients with certain eye diseases, such as dry age-related macular degeneration (AMD). AMD is accompanied by progressive loss of retinal pigment epithelial (RPE) cells, which are crucial for survival of the overlying photoreceptor cells. As is the case for PD, fetal transplants have been performed to replace lost cells, paving the way for stem cell-based approaches (Algvere et al., 1997). That study also highlighted the importance of immunosuppression despite the fact that the eye is considered somewhat immunoprivileged. Robust xeno-free, defined, and scalable differentiation protocols for RPE and photoreceptor cells have been developed and have shown promise in animal models (Osakada et al., 2008; Vaajasaari et al., 2011; McGill et al., 2017; Plaza Reyes et al., 2020; Ribeiro et al., 2021). The first transplantation of ES cell-derived RPE cells in humans was carried out in 2012 in patients with dry AMD and Stargardt disease, a disease leading to macular degeneration in younger individuals (Schwartz et al., 2012). In a subsequent larger study, there was visual improvement in half of the patients but also some significant side effects, such as cataract and inflammation (Schwartz et al., 2015). The first autologous iPSC-based cell therapy trial for any disease was performed in 2014 aiming to treat neovascular AMD (Mandai et al., 2017) and was later followed by an alternative strategy with banked HLA-matched allogeneic iPSCs to reduce the need of immunosuppression (Sugita et al., 2020). An alternative strategy to minimize immunological rejection has recently been reported through removal of HLA class I and II (Petrus-Reurer et al., 2020). Although transplantation of PSC-derived RPE cells shows promise for potentially halting further progression of disease, restoration of vision will ultimately also require replacement of lost photoreceptors.

In type 1 diabetes, β-cells in the pancreas are lost, and although whole pancreas or islet transplantation provides relief from hypoglycemia, donor tissue is in limited supply (Krentz et al., 2021), making type 1 diabetes a candidate for cell therapy-based approaches. A first-generation protocol for ES or iPS cell differentiation to β-cells initially yielded mixed cellular phenotypes and no glucose-responding cells (D'Amour et al., 2005). Advanced protocols produced human β-cells that turned out to be glucose responding and insulin secreting but only after transplantation and further maturation in mice (Kroon et al., 2008). The company ViaCyte conducted a phase 1/2 clinical trial using these immature endocrine cells held in an encapsulation device, and although the cells were tolerated after transplantation, no evidence of insulin production was reported (Henry et al., 2018). The next step was to create holes in the encapsulation device to allow nutrients and oxygen exchange, and with this modification, now requiring the administration of immunosuppressants, a few of the 15 patients receiving the cells and device have shown evidence of insulin production (stimulated C-peptide levels) (Ramzy et al., 2021). The company Vertex, after having acquired Semma therapeutics, has taken a different approach. By use of stem cell-derived islets that are fully differentiated and mature (Pagliuca et al., 2014), positive results in blood glucose control and therapeutic levels of insulin production have been reported from the first patient transplanted with such cells (VX880) into the liver, again along with immunosuppressants.

Myocardial infarction leads to muscle loss and formation of fibrotic tissue, and there are currently no functional therapies to replace lost or ailing cardiomyocytes. Moreover, the heart appears to be one of the organs lacking a robust endogenous adult stem cell pool (Senyo et al., 2013). For two decades, various types of adult cells have therefore been transplanted to improve post-infarction heart function, but with rather modest success (see Murry and MacLellan, 2020 for review). Hopes have since been pinned instead on cell therapy using *in vitro*-engineered cardiomyocytes. Initially, it may have been thought that development of such therapies would be rather straightforward, given that ES cells easily could be differentiated into beating cardiomyocytes in the culture dish, an *in vitro* differentiation paradigm that has been used for disease-modeling of different channelopathies (Giacomelli et al., 2020). The *in vitro*-differentiated cardiomyocytes are, however, thus far immature and do not exhibit all the properties of a fully differentiated cardiomyocyte (Karbassi et al., 2020). This has hampered clinical testing, but engraftment in animal models provides reason for cautious optimism. Survival and engraftment of cardiomyocytes have been demonstrated in rats and guinea pigs (Riegler et al., 2015; Weinberger et al., 2016), and improved cardiac function following transplantation has been demonstrated in experimentally infarcted macaques (Liu et al., 2018) and pigs (Romagnuolo et al., 2019). Furthermore, microvascular grafts have been tested and shown to improve perfusion in infarcted rat hearts (Redd et al., 2019), and patches of iPS-derived cardiomyocytes, smooth muscle cells, and

endothelial cells to experimentally infarcted pig hearts resulted in improved ventricular function (Gao et al., 2018). The transplantation of ES/iPS-derived cardiomyocytes to pigs and non-human primates has however also caused arrythmias, presumably as a consequence of the transplanted patch acting as an ectopic pacemaker (Liu et al., 2018; Romagnuolo et al., 2019). Although a first attempt to transplant human ES cell-derived cardiomyocytes to a patient has been reported (Menasché et al., 2015), the arrythmias observed in animal experiments currently represent a serious concern, which will require further research to address.

THE INTERNATIONAL SOCIETY FOR STEM CELL RESEARCH

Early in the 21st century, the technology to generate human ES cells was established, and there was a debate in several countries, not least in the US, on how the use of, and generation of, novel, ES cell lines should be regulated. These questions were also discussed among stem cell scientists, but there was no organized international forum for exchanging ideas, showcasing the latest research results, and discussing the ethical implications of stem cells and their use in future therapy development. As a response to this, Leonard Zon started the International Society for Stem Cell Research (ISSCR), with the ambition to promote the science in the field but also to engage in public outreach and communication, advocacy, and policy—a choice that may seem obvious now but was not obvious then.

The overarching mission of ISSCR is the promotion of excellence in stem cell science and in translation of research for the benefit of human health. In 2003, ISSCR organized its first Annual Meeting, which since then has been an integral part of ISSCR's activities (for a timeline of milestone events in ISSCR, see Figure 2). The Annual Meetings have grown in size from around 500 participants during the first years to 3,000–4,000 participants from over 60 countries in recent years (see Figure S1 for a list of the Annual Meetings). The majority of the Annual Meetings have been held in the US but also span the globe, and to widen its geographical footprint and to complement the large-scale Annual Meeting with a more intimate meeting format, ISSCR launched so-called International Symposia (see Figure S1 for a list of all International Symposia). The first International Symposium was held in Shanghai in 2008, and over the years 18 physical and two virtual International Symposia, which accommodate 250–500 participants, have been held in nine countries. In 2015, the Workshop on Clinical Translation, as a part of the Annual Meeting, was established to broaden the interface to the translational and clinical community. Along similar lines, the "Stem Cells Clinical Trials: Practical Advice for Physicians and Ethics/Institutional Review Boards" was published in 2018.

In 2006, ISSCR took a bold step by publishing a first set of Guidelines for Stem Cell Research and Clinical Translation (hereafter called the Guidelines). Recognizing the need to lay out principles for how stem cell research should be conducted in an ethically sound way and with high research integrity, ISSCR published the first set of Guidelines in 2006. New versions of the Guidelines appeared in 2008, 2016, and 2021, and the focus of each version reflects where the field of stem cell research stood at the time and what technologies were emerging. The 2006 edition of the Guidelines thus placed a strong focus on human ES cells, whereas later versions have incorporated recommendations, for example, for iPS cell, organoid, blastoid, editing of the human genome, and embryo research and for interspecies chimera research. The Guidelines have been proactive in providing recommendations for emerging fields, for example by including recommendations for mitochondrial replacement techniques (MRT), a technology currently allowed only in the UK, in the 2021 edition. By providing principled recommendations for how research and its application should be conducted, and whether some of the currently widely accepted regulatory frameworks should be updated or altered, the Guidelines has, inevitably in some cases, stirred a debate in the stem cell community. One such example is the suggestion to modify the so-called 14-day limit for research on human embryos, which dates back to the 1980s (McLaren, 1984) and is enshrined in law in more than 10 countries (Cavaliere, 2017). The notion that human embryos could be sustained *in vitro* for up to 13 days after fertilization (Deglincerti et al., 2016; Shahbazi et al., 2016) triggered an interest in revisiting the 14-day limit (Hyun et al., 2021; McCully, 2021). The 2021 Guidelines calls for a broader discussion on extending the time limit for embryos in culture beyond 14 days under special circumstances and with appropriate oversight (Lovell-Badge et al., 2021; Master et al., 2021). The call to open discussion on the 14-day rule has been challenged by some scientists (Green et al., 2021; Johnston et al., 2021). Another area of discussion regards ISSCR's position on editing of the human germ line. Genomic editing for the germ line is currently prohibited in most countries, and the 2021 Guidelines agrees that clinical application should be prohibited at this time but recommends that research be supported. This has led to a debate, as some hold the view that the germ line should be sacrosanct and spared from genome-editing exercises altogether (Baylis, 2021).

As stem cell research and the marketing of unproven stem cell therapies entered the public's awareness, ISSCR developed information about stem cell research directly for the general public. In 2008, ISSCR published the

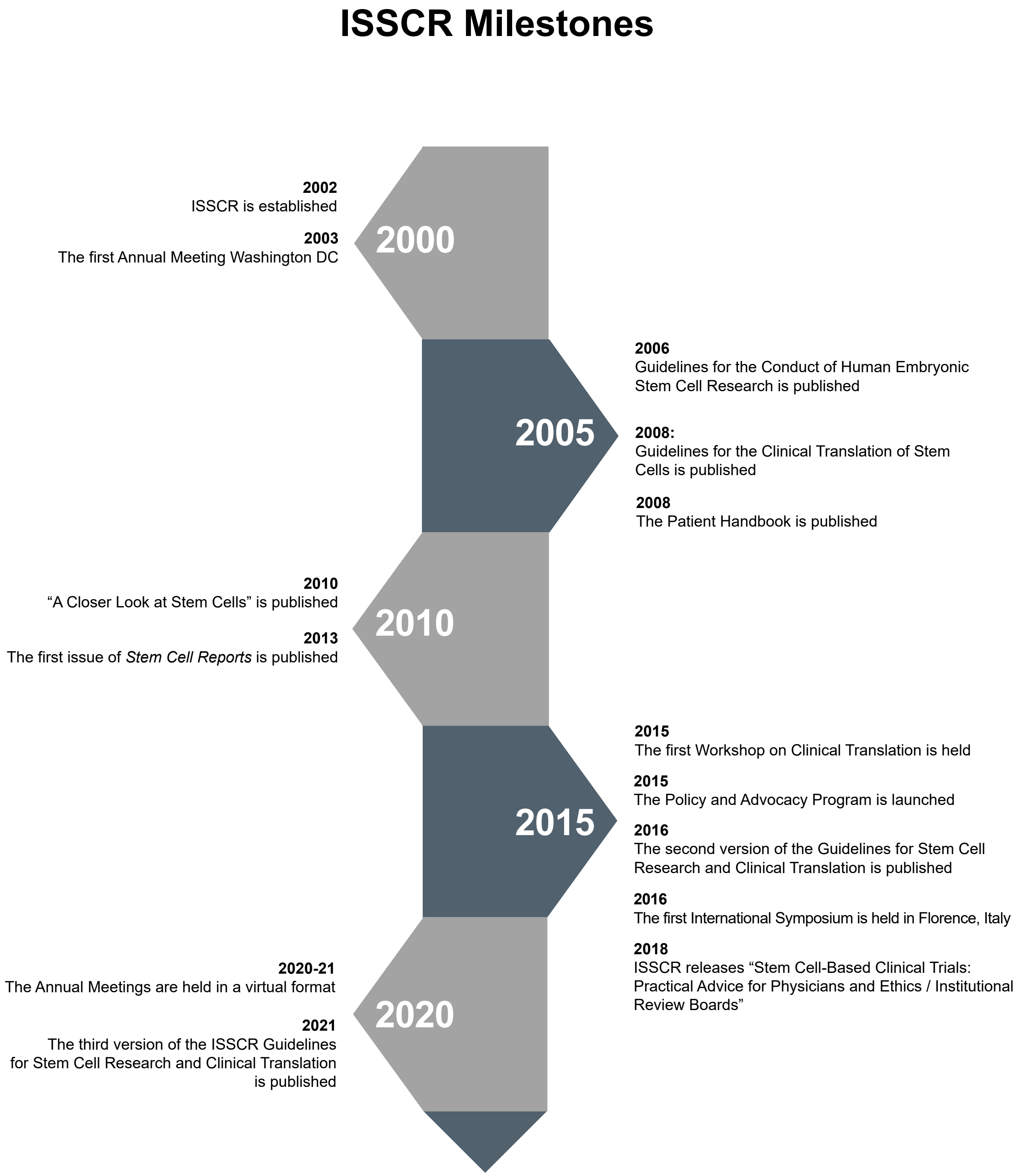

Figure 2. Time axis for ISSCR milestones

"Patient Handbook," which in 12 languages provides answers to frequently asked questions about stem cell therapy and has since been updated. Two years later, in 2010, another public education initiative was launched, when the "A Closer Look at Stem Cells" Website was introduced, an award-winning initiative to provide easy-to-grasp

information about many aspects of stem cell biology and its impact on human health, including fact-based information on stem cell therapies and so-called unproven (a.k.a. “snake oil”) stem cell-based therapies. ISSCR has been very active in terms of advocacy, notably by launching a policy and advocacy program in 2015, and by informing about the risks of unproven therapies. ISSCR members have on several occasions testified on behalf of ISSCR before governing bodies around the globe to support and defend important scientific principles and have provided expert testimony on issues such as ES cell and human fetal research, MRT, and unproven therapies.

In 2013, ISSCR took another major step, when the first issue of the journal *Stem Cell Reports* was published. *Stem Cell Reports*, which is published by Cell Press, Elsevier, has established itself as a leading journal in the field, currently publishing more than 200 articles per year and with a journal impact factor of 7.7 (2020). During its first 20 years, ISSCR has furthermore established several awards to recognize important scientific discoveries or other outstanding achievements by stem cell scientists (for a complete list of the ISSCR awards and awardees, see Figure S2). The Anne McLaren Memorial Award was established in 2008, followed by the Outstanding Young Investigator Award in 2009 (since 2018 called the Dr. Susan Lim Award for Outstanding Young Investigator), and the McEwen Award for Innovation (since 2018 called the ISSCR Award for Innovation) the Public Service Award and the Ernest McCulloch Memorial Lecture were established in 2010. In 2016, the Tobias Award Lecture was established, followed by the ISSCR Achievement Award and the Momentum Award in 2020 Figure S2.

ISSCR, like many other societies, was hit by the COVID-19 pandemic, and the 2020 and 2021 Annual Meetings needed to be switched from a physical to a digital meeting format. ISSCR, however, rapidly managed to gain the necessary expertise in arranging virtual meetings. The newly learned skills in digital content creation and meeting organization will also be useful going forward into the post-pandemic era, where a mix of real-life, hybrid, and virtual meetings will likely be the norm for ISSCR and many other societies. The ability to create professional virtual content is also increasingly used by ISSCR to produce courses, workshops, and activities such as ISSCR Digital. This contributes to a more dynamic, “year-round active” society, an improvement over the traditionally strong focus on the Annual Meeting with relatively few other activities spaced out throughout the rest of the year.

CONCLUDING REMARKS

During its first 20 years, ISSCR has made remarkable progress and established itself as a trusted voice for stem cell research, ethics, policy, and advocacy, and is providing an expanding portfolio of scientific meetings. ISSCR has also helped to call out unproven cell therapies and importantly published guidelines for how to conduct stem cell research with scientific and ethical integrity. With the current pace of progress in stem cell research, it will be interesting to see what new topics will be presented at future ISSCR meetings and addressed in new editions of the Guidelines. I, however, rest assured that ISSCR will be able to handle these tasks in a scholarly and wise manner. I wish ISSCR the best of luck in these future endeavors and a happy 20th birthday!

SUPPLEMENTAL INFORMATION

Supplemental information can be found online at https://doi.org/10.1016/j.stemcr.2022.04.004.

CONFLICTS OF INTEREST

U.L. holds a research grant from Merck KGaA but no personal remuneration. U.L. is a member of the Editorial Board of *Stem Cell Reports*.

ACKNOWLEDGMENTS

I thank several past and present members of the ISSCR Executive Board and members of the ISSCR staff for valuable comments on the manuscript text; all errors are my own. I apologize that not all work can be cited because of space limitations. Work in the author’s laboratory is supported by the Swedish Research Council and the Swedish Cancer Society.

REFERENCES

Algvere, P.V., Berglin, L., Gouras, P., Sheng, Y., and Kopp, E.D. (1997). Transplantation of RPE in age-related macular degeneration: observations in disciform lesions and dry RPE atrophy. Graefes Arch. Clin. Exp. Ophthalmol. *235*, 149–158.

Ali, N., Zirak, B., Rodriguez, R.S., Pauli, M.L., Truong, H.A., Lai, K., Ahn, R., Corbin, K., Lowe, M.M., Scharschmidt, T.C., et al. (2017). Regulatory T cells in skin facilitate epithelial stem cell differentiation. Cell *169*, 1119–1129.e11.

Altman, J. (1961). Are new neurons formed in the brains of adult mammals? Science *135*, 1127–1128.

Altman, J. (1969). Autoradiographic and histological studies of postnatal neurogenesis. IV. Cell proliferation and migration in the anterior forebrain, with special reference to persisting neurogenesis in the olfactory bulb. J. Comp. Neurol. *137*, 433–457.

Altman, J., and Das, G.D. (1965). Autoradiographic and histological evidence of postnatal hippocampal neurogenesis in rats. J. Comp. Neurol. *124*, 319–335.

Aragona, M., Dekoninck, S., Rulands, S., Lenglez, S., Mascré, G., Simons, B.D., and Blanpain, C. (2017). Defining stem cell dynamics and migration during wound healing in mouse skin epidermis. Nat. Commun. *8*, 14684.

Aragona, M., Sifrim, A., Malfait, M., Song, Y., van Herck, J., Dekoninck, S., Gargouri, S., Lapouge, G., Swedlund, B., Dubois, C.,

et al. (2020). Mechanisms of stretch-mediated skin expansion at single-cell resolution. Nature *584*, 268–273. https://doi.org/10.1038/s41586-020-2555-7.

Babu, R.O., Lui, V.C.H., Chen, Y., Yiu, R.S.W., Ye, Y., Niu, B., Wu, Z., Zhang, R., Yu, M.O.N., Chung, P.H.Y., et al. (2020). Beta-amyloid deposition around hepatic bile ducts is a novel pathobiological and diagnostic feature of biliary atresia. J. Hepatol. *73*, 1391–1403.

Bach, F.H., Albertini, R.J., Joo, P., Anderson, J.L., and Bortin, M.M. (1968). Bone-marrow transplantation in a patient with the Wiskott-Aldrich syndrome. Lancet *2*, 1364–1366.

Backlund, E.O., Granberg, P.O., Hamberger, B., Knutsson, E., Mårtensson, A., Sedvall, G., Seiger, Å., and Olson, L. (1985). Transplantation of adrenal medullary tissue to striatum in parkinsonism. First clinical trials. J. Neurosurg. *62*, 169–173.

Banks-Schlegel, S., and Green, H. (1980). Formation of epidermis by serially cultivated human epidermal cells transplanted as an epithelium to athymic mice. Transplantation *29*, 308–313.

Barker, N., van Es, J.H., Kuipers, J., Kujala, P., van den Born, M., Cozijnsen, M., Haegebarth, A., Korving, J., Begthel, H., Peters, P.J., et al. (2007). Identification of stem cells in small intestine and colon by marker gene Lgr5. Nature *449*, 1003–1007.

Barker, R.A., Drouin-Ouellet, J., and Parmar, M. (2015). Cell-based therapies for Parkinson disease-past insights and future potential. Nat. Rev. Neurol. *11*, 492–503.

Barker, R.A., Björklund, A., Frucht, S.J., and Svendsen, C.N. (2021). Stem cell-derived dopamine neurons: will they replace DBS as the leading neurosurgical treatment for Parkinson's disease? J. Parkinsons Dis. *11*, 909–917.

Barrandon, Y., and Green, H. (1987). Three clonal types of keratinocyte with different capacities for multiplication. Proc. Natl. Acad. Sci. U S A *84*, 2302–2306.

Baylis, F. (2021). ISSCR guidelines fudge heritable human-genome editing. Nature *594*, 333.

Becker, A.J., Mcculloch, E.A., and Till, J.E. (1963). Spleen colonies derived from transplanted mouse marrow cells. Nature *197*, 452–454.

Belmadani, A., Ren, D., Bhattacharyya, B.J., Rothwangl, K.B., Hope, T.J., Perlman, H., and Miller, R.J. (2015). Identification of a sustained neurogenic zone at the dorsal surface of the adult mouse hippocampus and its regulation by the chemokine SDF-1. Hippocampus *25*, 1224–1241.

Beumer, J., and Clevers, H. (2021). Cell fate specification and differentiation in the adult mammalian intestine. Nat. Rev. Mol. Cell Biol. *22*, 39–53.

Björklund, A., Dunnett, S.B., Stenevi, U., Lewis, M.E., and Iversen, S.D. (1980). Reinnervation of the denervated striatum by substantia nigra transplants: functional consequences as revealed by pharmacological and sensorimotor testing. Brain Res. *199*, 307–333.

Blanpain, C., and Fuchs, E. (2009). Epidermal homeostasis: a balancing act of stem cells in the skin. Nat. Rev. Mol. Cell Biol. *10*, 207–217.

Blanpain, C., Lowry, W.E., Geoghegan, A., Polak, L., and Fuchs, E. (2004). Self-renewal, multipotency, and the existence of two cell populations within an epithelial stem cell niche. Cell *118*, 635–648.

Boldrini, M., Fulmore, C.A., Tartt, A.N., Simeon, L.R., Pavlova, I., Poposka, V., Rosoklija, G.B., Stankov, A., Arango, V., Dwork, A.J., et al. (2018). Human hippocampal neurogenesis persists throughout aging. Cell Stem Cell *22*, 589–599.

Bonilla, S., Hall, A.C., Pinto, L., Attardo, A., Götz, M., Huttner, W.B., and Arenas, E. (2008). Identification of midbrain floor plate radial glia-like cells as dopaminergic progenitors. Glia *56*, 809–820.

Bordignon, C., Notarangelo, L.D., Nobili, N., Ferrari, G., Casorati, G., Panina, P., Mazzolari, E., Maggioni, D., Rossi, C., Servida, P., et al. (1995). Gene therapy in peripheral blood lymphocytes and bone marrow for ADA- immunodeficient patients. Science *270*, 470–475.

Bradley, A., Evans, M., Kaufman, M.H., and Robertson, E. (1984). Formation of germ-line chimaeras from embryo-derived teratocarcinoma cell lines. Nature *309*, 255–256.

Breunig, J.J., Haydar, T.F., and Rakic, P. (2011). Neural stem cells: historical perspective and future prospects. Neuron *70*, 614–625.

Briggs, R., and King, T.J. (1952). Transplantation of living nuclei from blastula cells into enucleated frogs' eggs. Proc. Natl. Acad. Sci. U S A *38*, 455–463.

Brinster, R.L. (1974). The effect of cells transferred into the mouse blastocyst on subsequent development. J. Exp. Med. *140*, 1049–1056.

Briscoe, J., Sussel, L., Serup, P., Hartigan-O'Connor, D., Jessell, T.M., Rubenstein, J.L.R., and Ericson, J. (1999). Homeobox gene Nkx2.2 and specification of neuronal identity by graded Sonic hedgehog signalling. Nature *398*, 622–627.

Buczacki, S.J.A., Zecchini, H.I., Nicholson, A.M., Russell, R., Vermeulen, L., Kemp, R., and Winton, D.J. (2013). Intestinal label-retaining cells are secretory precursors expressing lgr5. Nature *495*, 65–69.

Calvi, L.M., Adams, G.B., Weibrecht, K.W., Weber, J.M., Olson, D.P., Knight, M.C., Martin, R.P., Schipani, E., Divieti, P., Bringhurst, F.R., et al. (2003). Osteoblastic cells regulate the haematopoietic stem cell niche. Nature *425*, 841–846.

Castellana, D., Paus, R., and Perez-Moreno, M. (2014). Macrophages contribute to the cyclic activation of adult hair follicle stem cells. PLoS Biol. *12*, e1002002.

Cattaneo, E., and McKay, R. (1990). Proliferation and differentiation of neuronal stem cells regulated by nerve growth factor. Nature *347*, 762–765.

Cavaliere, G. (2017). A 14-day limit for bioethics: the debate over human embryo research. BMC Med. Ethics *18*, 38.

Chambers, I., Colby, D., Robertson, M., Nichols, J., Lee, S., Tweedie, S., and Smith, A. (2003). Functional expression cloning of Nanog, a pluripotency sustaining factor in embryonic stem cells. Cell *113*, 643–655.

Chambers, S.M., Fasano, C.A., Papapetrou, E.P., Tomishima, M., Sadelain, M., and Studer, L. (2009). Highly efficient neural conversion of human ES and iPS cells by dual inhibition of SMAD signaling. Nat. Biotechnol. *27*, 275–280.

Cheng, H., and Leblond, C.P. (1974). Origin, differentiation and renewal of the four main epithelial cell types in the mouse small intestine. V. Unitarian Theory of the origin of the four epithelial cell types. Am. J. Anat. *141*, 537–561.

Cieri, N., Maurer, K., and Wu, C.J. (2021). 60 years young: the evolving role of allogeneic hematopoietic stem cell transplantation in cancer immunotherapy. Cancer Res. *81*, 4373–4384.

Claudinot, S., Nicolas, M., Oshima, H., Rochat, A., and Barrandon, Y. (2005). Long-term renewal of hair follicles from clonogenic multipotent stem cells. Proc. Natl. Acad. Sci. U S A *102*, 14677–14682.

Clayton, E., Doupé, D.P., Klein, A.M., Winton, D.J., Simons, B.D., and Jones, P.H. (2007). A single type of progenitor cell maintains normal epidermis. Nature *446*, 185–189.

Cohen, I.G., Adashi, E.Y., Gerke, S., Palacios-Gonzaacutelez, C., and Ravitsky, V. (2020). The regulation of mitochondrial replacement techniques around the world. Annu. Rev. Genomics Hum. Genet. *21*, 565–586.

Comazzetto, S., Shen, B., and Morrison, S.J. (2021). Niches that regulate stem cells and hematopoiesis in adult bone marrow. Dev. Cell *56*, 1848–1860.

Cotsarelis, G., Sun, T.-T., and Lavker, R.M. (1990). Label-retaining cells reside in the bulge area of pilosebaceous unit: implications for follicular stem cells, hair cycle, and skin carcinogenesis. Cell *61*, 1329–1337.

D'Amour, K.A., Agulnick, A.D., Eliazer, S., Kelly, O.G., Kroon, E., and Baetge, E.E. (2005). Efficient differentiation of human embryonic stem cells to definitive endoderm. Nat. Biotechnol. *23*, 1534–1541.

Dantschakoff, W. (1908). Untersuchungen über die Entwickelung des Blutes und Bindegewebes bei den Vögeln'. Anat. Hefte *37*, 471–509.

Davis, A.A., and Temple, S. (1994). A self-renewing multipotential stem cell in embryonic rat cerebral cortex. Nature *372*, 263–266.

Deglincerti, A., Croft, G.F., Pietila, L.N., Zernicka-Goetz, M., Siggia, E.D., and Brivanlou, A.H. (2016). Self-organization of the in vitro attached human embryo. Nature *533*, 251–254.

Dekkers, J.F., Wiegerinck, C.L., de Jonge, H.R., Bronsveld, I., Janssens, H.M., de Winter-De Groot, K.M., Brandsma, A.M., de Jong, N.W.M., Bijvelds, M.J.C., Scholte, B.J., et al. (2013). A functional CFTR assay using primary cystic fibrosis intestinal organoids. Nat. Med. *19*, 939–945.

Dexter, T.M., Allen, T.D., and Lajtha, L.G. (1977). Conditions controlling the proliferation of haemopoietic stem cells in vitro. J. Cell Physiol. *91*, 335–344.

Ding, L., and Morrison, S.J. (2013). Haematopoietic stem cells and early lymphoid progenitors occupy distinct bone marrow niches. Nature *495*, 231–235.

Ding, L., Saunders, T.L., Enikolopov, G., and Morrison, S.J. (2012). Endothelial and perivascular cells maintain haematopoietic stem cells. Nature *481*, 457–462.

Doetsch, F., Caille, I., Lim, D.A., Garcı, J.M., and Alvarez-buylla, A. (1999). Subventricular zone astrocytes are neural stem cells in the adult mammalian brain. Cell *97*, 703–716.

Donati, G., Rognoni, E., Hiratsuka, T., Liakath-Ali, K., Hoste, E., Kar, G., Kayikci, M., Russell, R., Kretzschmar, K., Mulder, K.W., et al. (2017). Wounding induces dedifferentiation of epidermal Gata6 + cells and acquisition of stem cell properties. Nat. Cell Biol. *19*, 603–613.

Eiraku, M., Watanabe, K., Matsuo-Takasaki, M., Kawada, M., Yonemura, S., Matsumura, M., Wataya, T., Nishiyama, A., Muguruma, K., and Sasai, Y. (2008). Self-organized formation of polarized cortical tissues from ESCs and its active manipulation by extrinsic signals. Cell Stem Cell *3*, 519–532.

Eiraku, M., Takata, N., Ishibashi, H., Kawada, M., Sakakura, E., Okuda, S., Sekiguchi, K., Adachi, T., and Sasai, Y. (2011). Self-organizing optic-cup morphogenesis in three-dimensional culture. Nature *472*, 51–58.

Eriksson, P.S., Perrfilieva, E., BJörk-Eriksson, T., Alborn, A.-M., Nordborg, C., Peterson, D.A., and Gage, F.H. (1998). Neurogenesis in the adult human hippocampus. Nat. Med. *4*, 1313–1317.

Evans, M.J., and Kaufman, M.H. (1981). Establishment in culture of pluripotential cells from mouse embryos. Nature *292*, 154–156.

Falk, S., Han, D., and Karow, M. (2021). Cellular identity through the lens of direct lineage reprogramming. Curr. Opin. Genet. Dev. *70*, 97–103.

Ferrari, G., Thrasher, A.J., and Aiuti, A. (2021). Gene therapy using haematopoietic stem and progenitor cells. Nat. Rev. Genet. *22*, 216–234.

Fiore, V.F., Krajnc, M., Quiroz, F.G., Levorse, J., Pasolli, H.A., Shvartsman, S.Y., and Fuchs, E. (2020). Mechanics of a multilayer epithelium instruct tumour architecture and function. Nature *585*, 433–439.

Ford, C.E., Hamerton, J.L., Barnes, D.W.H., and Loutit, J.F. (1956). Cytological identification of radiation-chimæras. Nature *177*, 452–454.

Foudi, A., Hochedlinger, K., van Buren, D., Schindler, J.W., Jaenisch, R., Carey, V., and Hock, H. (2009). Analysis of histone 2B-GFP retention reveals slowly cycling hematopoietic stem cells. Nat. Biotechnol. *27*, 84–90.

Franjic, D., Skarica, M., Ma, S., Arellano, J.I., Tebbenkamp, A.T.N., Choi, J., Xu, C., Li, Q., Morozov, Y.M., Andrijevic, D., et al. (2022). Transcriptomic taxonomy and neurogenic trajectories of adult human, macaque, and pig hippocampal and entorhinal cells. Neuron *110*, 452–469.e14.

Freed, W.J., Morihisa, J.M., Spoor, E., Hoffer, B.J., Olson, L., Seiger, A., and Wyatt, R.J. (1981). Transplanted adrenal chromaffin cells in rat brain reduce lesion-induced rotational behaviour. Nature *292*, 351–352.

Fuchs, E., and Blau, H.M. (2020). Tissue stem cells: architects of their niches. Cell Stem Cell *27*, 532–556.

Fuchs, E., and Green, H. (1978). The expression of keratin genes in epidermis and cultured epidermal cells. Cell *15*, 887–897.

Fuchs, E., and Green, H. (1980). Changes in keratin gene expression during terminal differentiation of the keratinocyte. Cell *19*, 1033–1042.

Gao, L., Gregorich, Z.R., Zhu, W., Mattapally, S., Oduk, Y., Lou, X., Kannappan, R., Borovjagin, A.v., Walcott, G.P., Pollard, A.E., et al. (2018). Large cardiac muscle patches engineered from human induced-pluripotent stem cell-derived cardiac cells improve recovery from myocardial infarction in swine. Circulation *137*, 1712–1730.

Gatti, R.A., Meuwissen, H.J., Allen, H.D., Hong, R., and Good, R.A. (1968). Immunological reconstitution of sex-linked lymphopenic immunological deficiency. Lancet *2*, 1366–1369.

Ge, Y., Gomez, N.C., Adam, R.C., Nikolova, M., Yang, H., Verma, A., Lu, C.P.J., Polak, L., Yuan, S., Elemento, O., et al. (2017). Stem cell lineage infidelity drives wound repair and cancer. Cell *169*, 636–650.e14.

Genovese, P., Schiroli, G., Escobar, G., di Tomaso, T., Firrito, C., Calabria, A., Moi, D., Mazzieri, R., Bonini, C., Holmes, M.C., et al. (2014). Targeted genome editing in human repopulating haematopoietic stem cells. Nature *510*, 235–240.

Geurts, M.H., van der Vaart, J., Beumer, J., and Clevers, H. (2021). The organoid platform: promises and challenges as tools in the fight against COVID-19. Stem Cell Rep. *16*, 412–418.

Giacomelli, E., Meraviglia, V., Campostrini, G., Cochrane, A., Cao, X., van Helden, R.W.J., Krotenberg Garcia, A., Mircea, M., Kostidis, S., Davis, R.P., et al. (2020). Human-iPSC-Derived cardiac stromal cells enhance maturation in 3D cardiac microtissues and reveal non-cardiomyocyte contributions to heart disease. Cell Stem Cell *26*, 862–879.e11.

Gola, A., and Fuchs, E. (2021). Environmental control of lineage plasticity and stem cell memory. Curr. Opin. Cell Biol. *69*, 88–95.

Goldman, S.A., and Nottebohm, F. (1983). Neuronal production, migration, and differentiation in a vocal control nucleus of the adult female canary brain. Proc. Natl. Acad. Sci. U S A *80*, 2390–2394.

Gomez, C., Chua, W., Miremadi, A., Quist, S., Headon, D.J., and Watt, F.M. (2013). The interfollicular epidermis of adult mouse tail comprises two distinct cell lineages that are differentially regulated by Wnt, Edaradd, and Lrig1. Stem Cell Rep. *1*, 19–27.

Green, H., Kehinde, O., and Thomas, J. (1979). Growth of cultured human epidermal cells into multiple epithelia suitable for grafting. Proc. Natl. Acad. Sci. U S A *76*, 5665–5668.

Green, R.M., West, M.D., and Hayflick, L. (2021). Don't abandon 14-day limit on embryo research. Nature *594*, 333.

Gur-Cohen, S., Yang, H., Baksh, S.C., Miao, Y., Levorse, J., Kataru, R.P., Liu, X., de la Cruz-Racelis, J., Mehrara, B.J., and Fuchs, E. (2019). Stem cell–driven lymphatic remodeling coordinates tissue regeneration. Science *366*, 1218–1225.

Gurdon, J. (1962). The developmental capacity of nuclei taken from intestinal epithelium cells of feeding tadpoles. J. Embryol. Exp. Morphol. *10*, 622–640.

Haeckel, E. (1877). Anthropogenie oder Entwickelungsgeschichte des Menschen (Leipzig, W. Engelmann).

Haecker, V. (1892). Die Kerntheilungsvorgänge bei der Mesoderm- und Entodermbildung von Cyclops. Arch. Mikrosk. Anat. *39*, 556–581.

Henry, R.R., Pettus, J., Wilenxky, J., Shapiro, A.M.J., Senior, P.A., Roep, B., Wang, R., Kroon, E.J., Scott, M., D'Amour, K., et al. (2018). Initial clinical evaluation of VC-01TM combination product—a stem cell–derived islet replacement for type 1 diabetes (T1D). Diabetes *67*, 138-OR.

Hérault, A., Binnewies, M., Leong, S., Calero-Nieto, F.J., Zhang, S.Y., Kang, Y.A., Wang, X., Pietras, E.M., Chu, S.H., Barry-Holson, K., et al. (2017). Myeloid progenitor cluster formation drives emergency and leukaemic myelopoiesis. Nature *544*, 53–58.

Hirsch, T., Rothoeft, T., Teig, N., Bauer, J.W., Pellegrini, G., de Rosa, L., Scaglione, D., Reichelt, J., Klausegger, A., Kneisz, D., et al. (2017). Regeneration of the entire human epidermis using transgenic stem cells. Nature *551*, 327–332.

His, W. (1874). Unsere Körperform und das Physiologische Problem ihrer Entstehung. Briefe an einen Befreundeten Naturforscher (Engelmann).

Howe, S.J., Mansour, M.R., Schwarzwaelder, K., Bartholomae, C., Hubank, M., Kempski, H., Brugman, M.H., Pike-Overzet, K., Chatters, S.J., de Ridder, D., et al. (2008). Insertional mutagenesis combined with acquired somatic mutations causes leukemogenesis following gene therapy of SCID-X1 patients. J. Clin. Invest. *118*, 3143–3150.

Hsu, Y.C., Pasolli, H.A., and Fuchs, E. (2011). Dynamics between stem cells, niche, and progeny in the hair follicle. Cell *144*, 92–105.

Hsu, Y.C., Li, L., and Fuchs, E. (2014). Transit-amplifying cells orchestrate stem cell activity and tissue regeneration. Cell *157*, 935–949.

Huch, M., Dorrell, C., Boj, S.F., van Es, J.H., Li, V.S.W., van de Wetering, M., Sato, T., Hamer, K., Sasaki, N., Finegold, M.J., et al. (2013). In vitro expansion of single Lgr5+ liver stem cells induced by Wnt-driven regeneration. Nature *494*, 247–250.

Huch, M., Gehart, H., Boxtel, R., van Hamer, K., Blokzijl, F., Verstegen, M.M., Ellis, E., Wenum, M.V., Fuchs, S.A., Ligt, J.D., et al. (2015). Long-term culture of genome-stable bipotent stem cells from adult human liver. Cell *160*, 299–312.

Hyun, I., Bredenoord, A.L., Briscoe, J., Klipstein, S., and Tan, T. (2021). Human embryo research beyond the primitive streak. Science *371*, 998–1000.

Jadhav, U., Saxena, M., O'Neill, N.K., Saadatpour, A., Yuan, G.C., Herbert, Z., Murata, K., and Shivdasani, R.A. (2017). Dynamic reorganization of chromatin accessibility signatures during dedifferentiation of secretory precursors into Lgr5+ intestinal stem cells. Cell Stem Cell *21*, 65–77.

Jensen, K.B., Collins, C.A., Nascimento, E., Tan, D.W., Frye, M., Itami, S., and Watt, F.M. (2009). Lrig1 expression defines a distinct multipotent stem cell population in mammalian epidermis. Cell Stem Cell *4*, 427–439.

Johansson, C.B., Momma, S., Clarke, D.L., Risling, M., Lendahl, U., and Frisén, J. (1999). Identification of a neural stem cell in the adult mammalian central nervous system. Cell *96*, 25–34.

Johnston, J., Baylis, F., and Greele, H.T. (2021). ISSCR: grave omission of age limit for embryo research. Nature *594*, 495.

Jones, P.H., and Watt, F.M. (1993). Separation of human epidermal stem cells from transit amplifying cells on the basis of differences in integrin function and expression. Cell *73*, 713–724.

Kadoshima, T., Sakaguchi, H., Nakano, T., Soen, M., Ando, S., Eiraku, M., and Sasai, Y. (2014). Self-organization of axial polarity, inside-out layer pattern, and species-specific progenitor dynamics in human ES cell-derived neocortex. Proc. Natl. Acad. Sci. U S A *110*, 20284–20289.

Kagawa, H., Javali, A., Khoei, H.H., Sommer, T.M., Sestini, G., Novatchkova, M., Scholte op Reimer, Y., Castel, G., Bruneau, A.,

Maenhoudt, N., et al. (2022). Human blastoids model blastocyst development and implantation. Nature *601*, 600–605.

Karbassi, E., Fenix, A., Marchiano, S., Muraoka, N., Nakamura, K., Yang, X., and Murry, C.E. (2020). Cardiomyocyte maturation: advances in knowledge and implications for regenerative medicine. Nat. Rev. Cardiol. *17*, 341–359.

Kastner, C., Hendricks, A., Deinlein, H., Hankir, M., Germer, C.T., Schmidt, S., and Wiegering, A. (2021). Organoid models for cancer research—from bed to bench side and back. Cancers *13*, 4812.

Katayama, Y., Battista, M., Kao, W.M., Hidalgo, A., Peired, A.J., Thomas, S.A., and Frenette, P.S. (2006). Signals from the sympathetic nervous system regulate hematopoietic stem cell egress from bone marrow. Cell *124*, 407–421.

Kefalopoulou, Z., Politis, M., Piccini, P., Mencacci, N., Bhatia, K., Jahanshahi, M., Widner, H., Rehncrona, S., Brundin, P., Björklund, A., et al. (2014). Long-term clinical outcome of fetal cell transplantation for Parkinson disease: two case reports. JAMA Neurol. *71*, 83–87.

Kempermann, G., Gage, F.H., Aigner, L., Song, H., Curtis, M.A., Thuret, S., Kuhn, H.G., Jessberger, S., Frankland, P.W., Cameron, H.A., et al. (2018). Human adult neurogenesis: evidence and remaining questions. Cell Stem Cell *23*, 25–30.

Kiel, M.J., Yilmaz, Ö.H., Iwashita, T., Yilmaz, O.H., Terhorst, C., and Morrison, S.J. (2005). SLAM family receptors distinguish hematopoietic stem and progenitor cells and reveal endothelial niches for stem cells. Cell *121*, 1109–1121.

Kiel, M.J., Radice, G.L., and Morrison, S.J. (2007). Lack of evidence that hematopoietic stem cells depend on N-Cadherin-Mediated adhesion to osteoblasts for their maintenance. Cell Stem Cell *1*, 204–217.

Kikuchi, T., Morizane, A., Doi, D., Magotani, H., Onoe, H., Hayashi, T., Mizuma, H., Takara, S., Takahashi, R., Inoue, H., et al. (2017). Human iPS cell-derived dopaminergic neurons function in a primate Parkinson's disease model. Nature *548*, 592–596.

Kim, T.W., Koo, S.Y., and Studer, L. (2020). Pluripotent stem cell therapies for Parkinson disease: present challenges and future opportunities. Front. Cell Dev. Biol. *8*, 729.

Kirkeby, A., Nolbrant, S., Tiklova, K., Heuer, A., Kee, N., Cardoso, T., Ottosson, D.R., Lelos, M.J., Rifes, P., Dunnett, S.B., et al. (2017). Predictive markers guide differentiation to improve graft outcome in clinical translation of hESC-based therapy for Parkinson's disease. Cell Stem Cell *20*, 135–148.

Kleinsmith, L.J., and Pierce, G.B. (1964). Multipotentiality of single embryonal carcinoma cells. Cancer Res. *24*, 1544–1551.

Kordower, J.H., Chu, Y., Hauser, R.A., Freeman, T.B., and Olanow, C.W. (2008). Lewy body-like pathology in long-term embryonic nigral transplants in Parkinson's disease. Nat. Med. *14*, 504–506.

Krentz, N.A.J., Shea, L.D., Huising, M.O., and Shaw, J.A.M. (2021). Restoring normal islet mass and function in type 1 diabetes through regenerative medicine and tissue engineering. Lancet Diabetes Endocrinol. *9*, 708–724.

Kriks, S., Shim, J.W., Piao, J., Ganat, Y.M., Wakeman, D.R., Xie, Z., Carrillo-Reid, L., Auyeung, G., Antonacci, C., Buch, A., et al. (2011). Dopamine neurons derived from human ES cells efficiently engraft in animal models of Parkinson's disease. Nature *480*, 547–551.

Kroon, E., Martinson, L.A., Kadoya, K., Bang, A.G., Kelly, O.G., Eliazer, S., Young, H., Richardson, M., Smart, N.G., Cunningham, J., et al. (2008). Pancreatic endoderm derived from human embryonic stem cells generates glucose-responsive insulin-secreting cells in vivo. Nat. Biotechnol. *26*, 443–452.

Lancaster, M.A., and Knoblich, J.A. (2014). Organogenesisin a dish: modeling development and disease using organoid technologies. Science *345*, 283–292.

Lancaster, M.A., Renner, M., Martin, C.A., Wenzel, D., Bicknell, L.S., Hurles, M.E., Homfray, T., Penninger, J.M., Jackson, A.P., and Knoblich, J.A. (2013). Cerebral organoids model human brain development and microcephaly. Nature *501*, 373–379.

Lassar, A.B., Paterson, B.M., and Weintraub, H. (1986). Transfection of a DNA locus that mediates the conversion of 10T12 fibroblasts to myoblasts. Cell *47*, 649–656.

Lechler, T., and Fuchs, E. (2005). Asymmetric cell divisions promote stratification and differentiation of mammalian skin. Nature *437*, 275–280.

Li, J.Y., Englund, E., Holton, J.L., Soulet, D., Hagell, P., Lees, A.J., Lashley, T., Quinn, N.P., Rehncrona, S., Björklund, A., et al. (2008). Lewy bodies in grafted neurons in subjects with Parkinson's disease suggest host-to-graft disease propagation. Nat. Med. *14*, 501–503.

Li, R., Zhong, C., and Izpisua Belmonte, J.C. (2022). Time matters: human blastoids resemble the sequence of blastocyst development. Cell *185*, 581–584.

Liem, K.F., Tremml, G., and Jessell, T.M. (1997). A role for the roof plate and its resident TGFβ-related proteins in neuronal patterning in the dorsal spinal cord. Cell *91*, 127–138.

Lindvall, O., Rehncrona, S., Brundin, P., Gustavii, B., Åstedt, B., Widner, H., Lindholm, T., Björklund, A., Leenders, K.L., Rothwell, J.C., et al. (1989). Human fetal dopamine neurons grafted into the striatum in two patients with severe Parkinson's disease. Arch. Neurol. *46*, 615–631.

Liu, Y.W., Chen, B., Yang, X., Fugate, J.A., Kalucki, F.A., Futakuchi-Tsuchida, A., Couture, L., Vogel, K.W., Astley, C.A., Baldessari, A., et al. (2018). Human embryonic stem cell-derived cardiomyocytes restore function in infarcted hearts of non-human primates. Nat. Biotechnol. *36*, 597–605.

Lopez-Garcia, C., Klein, A.M., Simons, B.D., and Winton, D.J. (2010). Intestinal stem cell replacement follows a pattern of neutral drift. Science *330*, 822–825.

Lovell-Badge, R., Anthony, E., Barker, R.A., Bubela, T., Brivanlou, A.H., Carpenter, M., Charo, R.A., Clark, A., Clayton, E., Cong, Y., et al. (2021). ISSCR guidelines for stem cell research and clinical translation: the 2021 update. Stem Cell Rep. *16*, 1398–1408.

Lown, R.N., Philippe, J., Navarro, W., van Walraven, S.M., Philips-Johnson, L., Fechter, M., Pawson, R., Bengtsson, M., Beksac, M., Field, S., et al. (2014). Unrelated adult stem cell donor medical suitability: recommendations from the world marrow donor association clinical working group committee. Bone Marrow Transplant. *49*, 880–886.

Maehle, A.-H. (2011). Ambiguous cells: the emergence of the stem cell concept in the nineteenth and twentieth centuries. Notes Rec. R. Soc. Lond. *65*, 359–378.

Malatesta, P., Hartfuss, E., and Götz, M. (2000). Isolation of radial glial cells by fluorescent-activated cell sorting reveals a neuronal lineage. Development *127*, 5253–5263.

Mandai, M., Kurimoto, Y., and Takahashi, M. (2017). Autologous induced stem-cell–derived retinal cells for macular degeneration. N. Engl. J. Med. *377*, 792–793.

Martin, G.R. (1981). Isolation of a pluripotent cell line from early mouse embryos cultured in medium conditioned by teratocarcinoma stem cells. Proc. Natl. Acad. Sci. U S A *78*, 7634–7638.

Mascré, G., Dekoninck, S., Drogat, B., Youssef, K.K., Brohée, S., Sotiropoulou, P.A., Simons, B.D., and Blanpain, C. (2012). Distinct contribution of stem and progenitor cells to epidermal maintenance. Nature *489*, 257–262.

Master, Z., Lovell-Badge, R., and Knoppers, B. (2021). ISSCR upholds right to science. Nature *595*, 494.

Mathé, G., Amiel, J.L., Schwarzenberg, L., Cattan, A., Schneider, M., Devries, M.J., Tubiana, M., Lalanne, C., Binet, J.L., Papiernik, M., et al. (1965). Successful allogenic bone marrow transplantation in man: chimerism, induced specific tolerance and possible anti-leukemic effects. Blood *25*, 179–196.

Maximow, A. (1909). Der Lymphozyt als gemeinsame Stammzelle der verschiedenen Blutelemente in der embryonalen Entwicklung und im postfetalen Leben der Säugetiere. Fol. Hematol. *8*, 125–134.

McCarthy, N., Kraiczy, J., and Shivdasani, R.A. (2020). Cellular and molecular architecture of the intestinal stem cell niche. Nat. Cell Biol. *22*, 1033–1041.

McCully, S. (2021). The time has come to extend the 14-day limit. J. Med. Ethics *47*, e66.

McGill, T.J., Bohana-Kashtan, O., Stoddard, J.W., Andrews, M.D., Pandit, N., Rosenberg-Belmaker, L.R., Wiser, O., Matzrafi, L., Banin, E., Reubinoff, B., et al. (2017). Matzrafi, L., Banin, E., Reubinoff, B., et al. (2017). Long-term efficacy of GMP grade xeno-free hESC-derived RPE cells following transplantation. Transl. Vis. Sci. Technol. *6*, 17. https://doi.org/10.1167/tvst.6.3.17.

McLaren, A. (1984). Where to draw the line? Proc. R. Inst. *56*, 101–121.

Menasché, P., Vanneaux, V., Hagège, A., Bel, A., Cholley, B., Cacciapuoti, I., Parouchev, A., Benhamouda, N., Tachdjian, G., Tosca, L., et al. (2015). Human embryonic stem cell-derived cardiac progenitors for severe heart failure treatment: first clinical case report. Eur. Heart J. *36*, 2011–2017.

Mirzadeh, Z., Merkle, F.T., Soriano-Navarro, M., Garcia-Verdugo, J.M., and Alvarez-Buylla, A. (2008). Neural stem cells confer unique pinwheel architecture to the ventricular surface in neurogenic regions of the adult brain. Cell Stem Cell *3*, 265–278.

Morrison, S.J., and Scadden, D.T. (2014). The bone marrow niche for haematopoietic stem cells. Nature *505*, 327–334.

Morrison, S.J., and Weissman, I.L. (1994). The long-term repopulating subset of hematopoietic stem cells is deterministic and isolatable by phenotype. Immunity *1*, 661–673.

Murry, C.E., and MacLellan, W.R. (2020). Stem cells and the heart - the road ahead. Science *367*, 854–855.

Naik, S., Larsen, S.B., Gomez, N.C., Alaverdyan, K., Sendoel, A., Yuan, S., Polak, L., Kulukian, A., Chai, S., and Fuchs, E. (2017). Inflammatory memory sensitizes skin epithelial stem cells to tissue damage. Nature *550*, 475–480.

Navarro Negredo, P., Yeo, R.W., and Brunet, A. (2020). Aging and rejuvenation of neural stem cells and their niches. Cell Stem Cell *27*, 202–223.

Neumann, E. (1868). Über die bedeutung de knochenmarkers für die blutbildung. Zentralbl. Med. Wissensch. *6*, 689.

Nichols, J., Zevnik, B., Anastassiadis, K., Niwa, H., Klewe-Nebenius, D., Chambers, I., Schöler, H., and Smith, A. (1998). Formation of pluripotent stem cells in the mammalian embryo depends on the POU transcription factor Oct4. Cell *95*, 379–391.

Noctor, S.C., Flint, A.C., Weissman, T.A., Dammerman, R.S., and Kriegstein, A.R. (2001). Neurons derived from radial glial cells establish radial units in neocortex. Nature *409*, 714–720.

O'Connor, N.E., Mulliken, J.B., Banks-Schlegel, S., Kehinde, O., and Green, H. (1981). Grafting of burns with cultured epithelium prepared from autologous epidermal cells. Lancet *317*, 75–78.

Oguro, H., Ding, L., and Morrison, S.J. (2013). SLAM family markers resolve functionally distinct subpopulations of hematopoietic stem cells and multipotent progenitors. Cell Stem Cell *13*, 102–116.

Olson, L., and Seiger, Å. (1973). Development and growth of immature monoamine neurons in rat and man in situ and following intraocular transplantation in the rat. Brain Res. *62*, 353–360.

Ono, Y., Nakatani, T., Sakamoto, Y., Mizuhara, E., Minaki, Y., Kumai, M., Hamaguchi, A., Nishimura, M., Inoue, Y., Hayashi, H., et al. (2007). Differences in neurogenic potential in floor plate cells along an anteroposterior location: midbrain dopaminergic neurons originate from mesencephalic floor plate cells. Development *134*, 3213–3225.

Osakada, F., Ikeda, H., Mandai, M., Wataya, T., Watanabe, K., Yoshimura, N., Akaike, A., Sasai, Y., and Takahashi, M. (2008). Toward the generation of rod and cone photoreceptors from mouse, monkey and human embryonic stem cells. Nat. Biotechnol. *26*, 215–224.

Page, M.E., Lombard, P., Ng, F., Göttgens, B., and Jensen, K.B. (2013). The epidermis comprises autonomous compartments maintained by distinct stem cell populations. Cell Stem Cell *13*, 471–482.

Pagliuca, F.W., Millman, J.R., Gürtler, M., Segel, M., van Dervort, A., Ryu, J.H., Peterson, Q.P., Greiner, D., and Melton, D.A. (2014). Generation of functional human pancreatic β cells in vitro. Cell *159*, 428–439.

Pappenheim, A. (1896). Über entwickelung and ausbildung der erythroblasten. Virchows Arch. A Pathol. Anat. *145*, 587–643.

Paredes, M.F., Sorrells, S.F., Cebrian-Silla, A., Sandoval, K., Qi, D., Kelley, K.W., James, D., Mayer, S., Chang, J., Auguste, K.I., et al. (2018). Does adult neurogenesis persist in the human Hippocampus? Cell Stem Cell *23*, 780–781.

Park, S., Gonzalez, D.G., Guirao, B., Boucher, J.D., Cockburn, K., Marsh, E.D., Mesa, K.R., Brown, S., Rompolas, P., Haberman, A.M., et al. (2017). Tissue-scale coordination of cellular behaviour promotes epidermal wound repair in live mice. Nat. Cell Biol. *19*, 155–163.

Parmar, M., and Björklund, A. (2020). From skin to brain: a Parkinson's disease patient transplanted with his own cells. Cell Stem Cell *27*, 8–10.

Perlow, M.J., Freed, W.J., Hoffer, B.J., Seiger, A., Olson, L., and Wyatt, R.J. (1979). Brain grafts reduce motor abnormalities produced by destruction of nigrostriatal dopamine system. Science *204*, 643–647.

Petrus-Reurer, S., Winblad, N., Kumar, P., Gorchs, L., Chrobok, M., Wagner, A.K., Bartuma, H., Lardner, E., Aronsson, M., Plaza Reyes, Á., et al. (2020). Generation of retinal pigment epithelial cells derived from human embryonic stem cells lacking human leukocyte antigen class I and II. Stem Cell Rep. *14*, 648–662.

Plaza Reyes, A., Petrus-Reurer, S., Padrell Sánchez, S., Kumar, P., Douagi, I., Bartuma, H., Aronsson, M., Westman, S., Lardner, E., André, H., et al. (2020). Identification of cell surface markers and establishment of monolayer differentiation to retinal pigment epithelial cells. Nat. Commun. *11*, 1609. https://doi.org/10.1038/s41467-020-15326-5.

Potten, C.S., Owen, G., and Booth, D. (2002). Intestinal stem cells protect their genome by selective segregation of template DNA strands. J. Cell Sci. *115*, 2381–2388.

Price, J., and Thurlow, L. (1988). Cell lineage in the rat cerebral cortex: a study using retroviral-mediated gene transfer. Development *104*, 473–482.

Qian, X., Nguyen, H.N., Song, M.M., Hadiono, C., Ogden, S.C., Hammack, C., Yao, B., Hamersky, G.R., Jacob, F., Zhong, C., et al. (2016). Brain-region-specific organoids using mini-bioreactors for modeling ZIKV exposure. Cell *165*, 1238–1254.

Radakovits, R., Barros, C.S., Belvindrah, R., Patton, B., and Müller, U. (2009). Regulation of radial glial survival by signals from the meninges. J. Neurosci. *29*, 7694–7705.

Ramalho-Santos, M., and Willenbring, H. (2007). On the origin of the term "stem cell". Cell Stem Cell *1*, 35–38.

Ramzy, A., Thompson, D.M., Ward-Hartstonge, K.A., Ivison, S., Cook, L., Garcia, R.v., Loyal, J., Kim, P.T.W., Warnock, G.L., Levings, M.K., et al. (2021). Implanted pluripotent stem-cell-derived pancreatic endoderm cells secrete glucose-responsive C-peptide in patients with type 1 diabetes. Cell Stem Cell *28*, 2047–2061.e5.

Redd, M.A., Zeinstra, N., Qin, W., Wei, W., Martinson, A., Wang, Y., Wang, R.K., Murry, C.E., and Zheng, Y. (2019). Patterned human microvascular grafts enable rapid vascularization and increase perfusion in infarcted rat hearts. Nat. Commun. *10*, 584.

Rheinwald, J.G., and Green, H. (1975). Formation of a keratinizing epithelium in culture by a cloned cell line derived from a teratoma. Cell *6*, 317–330.

Ribeiro, J., Procyk, C.A., West, E.L., Pearson, R.A., Gonzalez-cordero, A., Ali, R.R., Ribeiro, J., Procyk, C.A., West, E.L., Hara-wright, M.O., et al. (2021). Restoration of visual function in advanced disease after transplantation of purified human pluripotent stem cell-derived cone photoreceptors. Cell Rep. *35*, 109022. https://doi.org/10.1016/j.celrep.2021.109022.

Rideout, W.M., Hochedlinger, K., Kyba, M., Daley, G.Q., and Jaenisch, R. (2002). Correction of a genetic defect by nuclear transplantation and combined cell and gene therapy. Cell *109*, 17–27.

Riegler, J., Tiburcy, M., Ebert, A., Tzatzalos, E., Raaz, U., Abilez, O.J., Shen, Q., Kooreman, N.G., Neofytou, E., Chen, V.C., et al. (2015). Human engineered heart muscles engraft and survive long term in a rodent myocardial infarction model. Circ. Res. *117*, 720–730.

Rivron, N.C., Frias-Aldeguer, J., Vrij, E.J., Boisset, J.C., Korving, J., Vivié, J., Truckenmüller, R.K., van Oudenaarden, A., van Blitterswijk, C.A., and Geijsen, N. (2018). Blastocyst-like structures generated solely from stem cells. Nature *557*, 106–111.

Rognoni, E., and Watt, F.M. (2018). Skin cell heterogeneity in development, wound healing, and cancer. Trends Cell Biol. *28*, 709–722.

Romagnuolo, R., Masoudpour, H., Porta-Sánchez, A., Qiang, B., Barry, J., Laskary, A., Qi, X., Massé, S., Magtibay, K., Kawajiri, H., et al. (2019). Human embryonic stem cell-derived cardiomyocytes regenerate the infarcted pig heart but induce ventricular tachyarrhythmias. Stem Cell Rep. *12*, 967–981.

Sato, T., Vries, R.G., Snippert, H.J., van de Wetering, M., Barker, N., Stange, D.E., van Es, J.H., Abo, A., Kujala, P., Peters, P.J., et al. (2009). Single Lgr5 stem cells build crypt-villus structures in vitro without a mesenchymal niche. Nature *459*, 262–265.

Sato, T., van Es, J.H., Snippert, H.J., Stange, D.E., Vries, R.G., van den Born, M., Barker, N., Shroyer, N.F., van de Wetering, M., and Clevers, H. (2011). Paneth cells constitute the niche for Lgr5 stem cells in intestinal crypts. Nature *469*, 415–418.

Schepers, A.G., Vries, R., van den Born, M., van de Wetering, M., and Clevers, H. (2011). Lgr5 intestinal stem cells have high telomerase activity and randomly segregate their chromosomes. EMBO J. *30*, 1104–1109.

Schofield, R. (1978). The relationship between the spleen colony-forming cell and the haemopoietic stem cell. Blood Cells *4*, 7–25.

Schwartz, S.D., Hubschman, J.P., Heilwell, G., Franco-Cardenas, V., Pan, C.K., Ostrick, R.M., Mickunas, E., Gay, R., Klimanskaya, I., and Lanza, R. (2012). Embryonic stem cell trials for macular degeneration: a preliminary report. Lancet *379*, 713–720.

Schwartz, S.D., Regillo, C.D., Lam, B.L., Eliott, D., Rosenfeld, P.J., Gregori, N.Z., Hubschman, J.-P., Davis, J.L., Heilwell, G., Spirn, M., et al. (2015). Human embryonic stem cell-derived retinal pigment epithelium in patients with age-related macular degeneration and Stargardt's macular dystrophy: follow-up of two open-label phase 1/2 studies. Lancet *385*, 509–516.

Schweitzer, J.S., Song, B., Herrington, T.M., Park, T.-Y., Lee, N., Ko, S., Jeon, J., Cha, Y., Kim, K., Li, Q., et al. (2020). Personalized iPSC-derived dopamine progenitor cells for Parkinson's disease. N. Engl. J. Med. *382*, 1926–1932.

Segel, M., Neumann, B., Hill, M.F.E., Weber, I.P., Viscomi, C., Zhao, C., Young, A., Agley, C.C., Thompson, A.J., Gonzalez, G.A., et al. (2019). Niche stiffness underlies the ageing of central nervous system progenitor cells. Nature *573*, 130–134.

Senyo, S.E., Steinhauser, M.L., Pizzimenti, C.L., Yang, V.K., Cai, L., Wang, M., Wu, T.D., Guerquin-Kern, J.L., Lechene, C.P., and Lee,

R.T. (2013). Mammalian heart renewal by pre-existing cardiomyocytes. Nature *493*, 433–436.

Severe, N., Karabacak, N.M., Gustafsson, K., Baryawno, N., Courties, G., Kfoury, Y., Kokkaliaris, K.D., Rhee, C., Lee, D., Scadden, E.W., et al. (2019). Stress-induced changes in bone marrow stromal cell populations revealed through single-cell protein expression mapping. Cell Stem Cell *25*, 570–583.

Shahbazi, M.N., Jedrusik, A., Vuoristo, S., Recher, G., Hupalowska, A., Bolton, V., Fogarty, N.M.E., Campbell, A., Devito, L.G., Ilic, D., et al. (2016). Self-organization of the human embryo in the absence of maternal tissues. Nat. Cell Biol. *18*, 700–708.

Shamblott, M.J., Axelman, J., Wang, S., Bugg, E.M., Littlefield, J.W., Donovan, P.J., Blumenthal, P.D., Huggins, G.R., and Gearhart, J.D. (1998). Derivation of pluripotent stem cells from cultured human primordial germ cells. Proc. Natl. Acad. Sci. U S A *95*, 13726–13731.

Shen, Q., Wang, Y., Kokovay, E., Lin, G., Chuang, S.M., Goderie, S.K., Roysam, B., and Temple, S. (2008). Adult SVZ stem cells lie in a vascular niche: a quantitative analysis of niche cell-cell interactions. Cell Stem Cell *3*, 289–300.

Shwartz, Y., Gonzalez-Celeiro, M., Chen, C.L., Pasolli, H.A., Sheu, S.H., Fan, S.M.Y., Shamsi, F., Assaad, S., Lin, E.T.Y., Zhang, B., et al. (2020). Cell types promoting goosebumps form a niche to regulate hair follicle stem cells. Cell *182*, 578–593.

Siegenthaler, J.A., Ashique, A.M., Zarbalis, K., Patterson, K.P., Hecht, J.H., Kane, M.A., Folias, A.E., Choe, Y., May, S.R., Kume, T., et al. (2009). Retinoic acid from the meninges regulates cortical neuron generation. Cell *139*, 597–609.

Silva-Vargas, V., Maldonado-Soto, A.R., Mizrak, D., Codega, P., and Doetsch, F. (2016). Age-dependent niche signals from the choroid plexus regulate adult neural stem cells. Cell Stem Cell *19*, 643–652.

Smart, I.H.M. (1970). Variation in the plane of cell cleavage during the process of stratification in the mouse epidermis. Br. J. Dermatol. *82*, 276–282.

Smart, I., and Leblond, C.P. (1961). Evidence for division and transformations of neuroglia cells in the mouse brain, as derived from radioautography after injection of thymidine-H3. J. Comp. Neurol. *116*, 349–367.

Snippert, H.J., van der Flier, L.G., Sato, T., van Es, J.H., van den Born, M., Kroon-Veenboer, C., Barker, N., Klein, A.M., van Rheenen, J., Simons, B.D., et al. (2010). Intestinal crypt homeostasis results from neutral competition between symmetrically dividing Lgr5 stem cells. Cell *143*, 134–144.

Soldner, F., and Jaenisch, R. (2018). Stem cells, genome editing, and the path to translational medicine. Cell *175*, 615–632.

Solter, D. (2006). From teratocarcinomas to embryonic stem cells and beyond: a history of embryonic stem cell research. Nat. Rev. Genet. *7*, 319–327.

Sorrells, S.F., Paredes, M.F., Cebrian-Silla, A., Sandoval, K., Qi, D., Kelley, K.W., James, D., Mayer, S., Chang, J., Auguste, K.I., et al. (2018). Human hippocampal neurogenesis drops sharply in children to undetectable levels in adults. Nature *555*, 377–381.

Spalding, K.L., Bhardwaj, R.D., Buchholz, B.A., Druid, H., and Frisén, J. (2005). Retrospective birth dating of cells in humans. Cell *122*, 133–143.

Spalding, K.L., Bergmann, O., Alkass, K., Bernard, S., Salehpour, M., Huttner, H.B., Boström, E., Westerlund, I., Vial, C., Buchholz, B.A., et al. (2013). Dynamics of hippocampal neurogenesis in adult humans. Cell *153*, 1219–1227.

Spangrude, G.J., Heimfeld, S., and Weissman, I.L. (1988). Purification and characterization of mouse hematopoietic stem cells. Science *241*, 58–62.

Spemann, H., and Mangold, H. (1924). Über induktion von embryonalanlagen durch implantation artfremder organisatoren. Arch. Mikrosk. Anat. Entwicklungsmech. *100*, 599–638.

Steptoe, P.C., Edwards, R.G., and Purdy, J.M. (1971). Human blastocysts grown in culture. Nature *229*, 132–133.

Stevens, L.C., and Little, C.C. (1954). Spontaneous testicular teratomas in an inbred strain of mice. Proc. Natl. Acad. Sci. U S A *40*, 1080–1087.

Sugita, S., Mandai, M., Hirami, Y., Takagi, S., Maeda, T., Fujihara, M., Matsuzaki, M., Yamamoto, M., Iseki, K., Hayashi, N., et al. (2020). HLA-matched allogeneic IPS cells-derived rpe transplantation for macular degeneration. J. Clin. Med. *9*, 2217.

Sugiyama, T., Kohara, H., Noda, M., and Nagasawa, T. (2006). Maintenance of the hematopoietic stem cell pool by CXCL12-CXCR4 chemokine signaling in bone marrow stromal cell niches. Immunity *25*, 977–988.

Sun, G.J., Zhou, Y., Ito, S., Bonaguidi, M.A., Stein-O'brien, G., Kawasaki, N.K., Modak, N., Zhu, Y., Ming, G.L., and Song, H. (2015). Latent tri-lineage potential of adult hippocampal neural stem cells revealed by Nf1 inactivation. Nat. Neurosci. *18*, 1722–1724.

Takagi, Y. (2016). History of neural stem cell research and its clinical application. Neurol. Med. Chir. *56*, 110–124.

Takahashi, J. (2020). iPS cell-based therapy for Parkinson's disease: a Kyoto trial. Regen. Ther. *13*, 18–22.

Takahashi, K., and Yamanaka, S. (2006). Induction of pluripotent stem cells from mouse embryonic and adult fibroblast cultures by defined factors. Cell *126*, 663–676.

Takahashi, K., Tanabe, K., Ohnuki, M., Narita, M., Ichisaka, T., Tomoda, K., and Yamanaka, S. (2007). Induction of pluripotent stem cells from adult human fibroblasts by defined factors. Cell *131*, 861–872.

Tavazoie, M., van der Veken, L., Silva-Vargas, V., Louissaint, M., Colonna, L., Zaidi, B., Garcia-Verdugo, J.M., and Doetsch, F. (2008). A specialized vascular niche for adult neural stem cells. Cell Stem Cell *3*, 279–288.

Tetteh, P.W., Basak, O., Farin, H.F., Wiebrands, K., Kretzschmar, K., Begthel, H., van den Born, M., Korving, J., de Sauvage, F., van Es, J.H., et al. (2016). Replacement of lost lgr5-positive stem cells through plasticity of their enterocyte-lineage daughters. Cell Stem Cell *18*, 203–213.

Thomas, E.D., Lochte, H.L., Jr., Lu, W.C., and Ferrebee, J.W. (1957). Intravenous infusion of bone marrow in patients receiving radiation and chemotherapy. N. Engl. J. Med. *257*, 951–957.

Thomson, J.A., Kalishman, J., Golos, T.G., Durning, M., Harris, C.P., Becker, R.A., and Hearn, J.P. (1995). Isolation of a primate embryonic stem cell line. Proc. Natl. Acad. Sci. U S A *92*, 7844–7848.

Thomson, J.A., Itskovitz-Eldor, J., Shapiro, S.S., Waknitz, M.A., Swiergiel, J.J., Marshall, V.S., and Jones, J.M. (1998). Embryonic stem cell lines derived from human blastocysts. Science *282*, 1145–1147.

Till, J.E., and McCulloch, E.A. (1961). A direct measurement of the radiation sensitivity of normal mouse bone marrow cells. Radiat. Res. *14*, 213–222.

Tumbar, T., Guasch, G., Greco, V., Blanpain, C., Lowry, W.E., Rendl, M., and Fuchs, E. (2004). Defining the epithelial stem cell niche in skin. Science *303*, 359–363.

Turner, D.L., and Cepko, C.C. (1987). A common progenitor for neurons and glia persists in rat retina late in development. Nature *328*, 131–136.

Ungerstedt, U. (1968). 6-Hydroxy-Dopamine induced degeneration of central monoamine neurons. Eur. J. Pharmacol. *5*, 107–110.

Vaajasaari, H., Ilmarinen, T., Juuti-Uusitalo, K., Rajala, K., Onnela, N., Narkilahti, S., Suuronen, R., Hyttinen, J., Uusitalo, H., and Skottman, H. (2011). Toward the defined and xeno-free differentiation of functional human pluripotent stem cell-derived retinal pigment epithelial cells. Mol. Vis. *17*, 558–575.

van Es, J.H., Sato, T., van de Wetering, M., Lyubimova, A., Yee Nee, A.N., Gregorieff, A., Sasaki, N., Zeinstra, L., van den Born, M., Korving, J., et al. (2012). Dll1 + secretory progenitor cells revert to stem cells upon crypt damage. Nat. Cell Biol. *14*, 1099–1104.

van Es, J.H., Wiebrands, K., López-Iglesias, C., van de Wetering, M., Zeinstra, L., van den Born, M., Korving, J., Sasaki, N., Peters, P.J., van Oudenaarden, A., et al. (2019). Enteroendocrine and tuft cells support Lgr5 stem cells on Paneth cell depletion. Proc. Natl. Acad. Sci. U S A *116*, 26599–26605.

Vierbuchen, T., Ostermeier, A., Pang, Z.P., Kokubu, Y., Südhof, T.C., and Wernig, M. (2010). Direct conversion of fibroblasts to functional neurons by defined factors. Nature *463*, 1035–1041.

Virchow, R.L.C. (1858). Die Cellularpathologie in Ihrer Begründung auf Physiologische und Pathologische Gewebelehre (Hirschwald).

Weinberger, F., Breckwoldt, K., Pecha, S., Kelly, A., Geertz, B., Starbatty, J., Yorgan, T., Cheng, K.H., Lessmann, K., Stolen, T., et al. (2016). Cardiac repair in Guinea pigs with human engineered heart tissue from induced pluripotent stem cells. Sci. Transl. Med. *8*, 363ra148.

Wilmut, I., Schnieke, A.E., McWhir, J., Kind, A.J., and Campbell, K.H.S. (1997). Viable offspring derived from fetal and adult mammalian cells. Nature *385*, 810–813.

Wilson, A., Laurenti, E., Oser, G., van der Wath, R.C., Blanco-Bose, W., Jaworski, M., Offner, S., Dunant, C.F., Eshkind, L., Bockamp, E., et al. (2008). Hematopoietic stem cells reversibly switch from dormancy to self-renewal during homeostasis and repair. Cell *135*, 1118–1129.

Wimmer, R.A., Leopoldi, A., Aichinger, M., Wick, N., Hantusch, B., Novatchkova, M., Taubenschmid, J., Hämmerle, M., Esk, C., Bagley, J.A., et al. (2019). Human blood vessel organoids as a model of diabetic vasculopathy. Nature *565*, 505–510.

Winton, D.J., Blount, M.A., and Ponder, B.A.J. (1988). A clonal marker induced by mutation in mouse intestinal epithelium. Nature *333*, 463–466.

Yanagida, A., Spindlow, D., Nichols, J., Dattani, A., Smith, A., and Guo, G. (2021). Naive stem cell blastocyst model captures human embryo lineage segregation. Cell Stem Cell *28*, 1016–1022.

Yang, N., Zuchero, J.B., Ahlenius, H., Marro, S., Ng, Y.H., Vierbuchen, T., Hawkins, J.S., Geissler, R., Barres, B.A., and Wernig, M. (2013). Generation of oligodendroglial cells by direct lineage conversion. Nat. Biotechnol. *31*, 434–439.

Yu, J., Vodyanik, M.A., Smuga-Otto, K., Antosiewicz-Bourget, J., Frane, J.L., Tian, S., Nie, J., Jonsdottir, G.A., Ruotti, V., Stewart, R., et al. (2007). Induced pluripotent stem cell lines derived from human somatic cells. Science *318*, 1917–1920.

Yu, V.W.C., Yusuf, R.Z., Oki, T., Wu, J., Saez, B., Wang, X., Cook, C., Baryawno, N., Ziller, M.J., Lee, E., et al. (2016). Epigenetic memory underlies cell-autonomous heterogeneous behavior of hematopoietic stem cells. Cell *167*, 1310–1322.

Yu, L., Wei, Y., Duan, J., Schmitz, D.A., Sakurai, M., Wang, L., Wang, K., Zhao, S., Hon, G.C., and Wu, J. (2021). Blastocyst-like structures generated from human pluripotent stem cells. Nature *591*, 1–7.

Yui, S., Nakamura, T., Sato, T., Nemoto, Y., Mizutani, T., Zheng, X., Ichinose, S., Nagaishi, T., Okamoto, R., Tsuchiya, K., et al. (2012). Functional engraftment of colon epithelium expanded in vitro from a single adult Lgr5 + stem cell. Nat. Med. *18*, 618–623.

Zhang, J., Niu, C., Ye, L., Huang, H., He, X., Tong, W.-G., Ross, J., Haug, J., Johnson, T., Feng, J.Q., et al. (2003). Identification of the haematopoietic stem cell niche and control of the niche size. Nature *425*, 837–841.

Zhang, B., Ma, S., Rachmin, I., He, M., Baral, P., Choi, S., Gonçalves, W.A., Shwartz, Y., Fast, E.M., Su, Y., et al. (2020). Hyperactivation of sympathetic nerves drives depletion of melanocyte stem cells. Nature *577*, 676–681.

Zhang, J., Wu, Q., Johnson, C.B., Pham, G., Kinder, J.M., Olsson, A., Slaughter, A., May, M., Weinhaus, B., D'Alessandro, A., et al. (2021). In situ mapping identifies distinct vascular niches for myelopoiesis. Nature *590*, 457–462.

Zhou, Q., Brown, J., Kanarek, A., Rajagopal, J., and Melton, D.A. (2008). In vivo reprogramming of adult pancreatic exocrine cells to b -cells. Nature *455*, 627–632.

Stem Cell Reports, Volume *17*

Supplemental Information

100 plus years of stem cell research—20 years of ISSCR

Urban Lendahl

ISSCR Annual Meetings and International Symposia

Annual Meetings:
2021 – virtual meeting because of the COVID-19 pandemic (originally planned to be held in Hamburg, Germany)
2020 – virtual meeting because of the COVD-19 pandemic (originally planned to be held in Boston, US)
2019 – Los Angeles, US
2018 – Melbourne, Australia
2017 – Boston, US
2016 – San Francisco, US
2015 – Stockholm, Sweden
2014 – Vancouver, Canada
2013 – Boston, US
2012 – Yokohama, Japan
2011 – Toronto, Canada
2010 – San Francisco, US
2009 – Barcelona, Spain
2008 – Philadelphia, US
2007 – Cairns, Australia
2006 – Toronto, Canada
2005 – San Francisco, US
2004 – Boston, US
2003 – Washington DC, US

International Symposia:
2021 – virtual meetings because of the COVID-19 pandemic
2020 – virtual meetings because of the COVID-19 pandemic
2019 – Amsterdam, the Netherlands
Seoul, South Korea
Toronto, Canada
2018 - Lausanne, Switzerland
2017 - Basel, Switzerland
Guangzhou, China
2016 - Dresden, Germany
Florence, Italy
Kyoto, Japan
2015 - Sozhou, China
2014 - San Diego, US
Singapore
2013 - Florence, Italy
Sozhou, China
2012 - Boston, US

San Francisco, US
Sao Paolo, Brazil
2011 - Sozhou, China
2010 - Amsterdam, the Netherlands
Boston, US
2009 - Buenos Aires, Argentina
2008 - Shanghai, China

Supplementary Figure 1:
A list of ISSCR Annual Meetings and International Symposia.

ISSCR Awards and Awardees

ISSCR Dr. Susan Lim Award for Outstanding Young Investigator (2009-2017 called the ISSCR Outstanding Young Investigator Award)

The ISSCR Dr. Susan Lim Award for Outstanding Young Investigator, supported by the Dr. Susan Lim Endowment for Education and Research Ltd., recognizes the exceptional achievements of an investigator in the early part of their independent career in stem cell research.

Awardees:
2021 - Madeline Lancaster
2020 - Allon M. Klein
2019 – Barbara Treutlein
2018 – Shuibing Chen
2017 – Jayaraj Rajagopal
2016 – Fernando Camargo
2015 – Paul Tesar
2014 – Valentina Greco
2013 – Marius Wernig
2012 – Cédric Blanpain
2011 – Robert Blelloch
2010 – Joanna Wysocka
2009 – Konrad Hochedlinger

ISSCR Achievement Award (introduced in 2020)

The ISSCR Achievement Award recognizes the transformative body of work of an investigator that has had a major impact on the field of stem cell research or regenerative medicine.

Awardees:
2021 – Janet Rossant

ISSCR Momentum Award (introduced in 2020)

The ISSCR Momentum Award recognizes the exceptional achievements of an investigator whose innovative research has established a major area of stem cell-related research with a strong trajectory for future success.

Awardees:
2021 – Valentina Greco

ISSCR Public Service Award (introduced in 2011)

The ISSCR Public Service Award is given in recognition of outstanding contributions of public service to the fields of stem cell research and regenerative medicine.

Awardees:
2021 – Robin Lovell-Badge
2020 – Susan L. Solomon
2019 – Eli and Edythe Broad
2018 – Megan Munsie
2017 – George Q. Daley
2015 – Alan Trounson
2014 – Paolo Bianco, Elena Cattaneo, Michele De Luca
2013 – Hiromitsu Ogawa, Betty Jean Crouch Ogawa
2012 – Rob and Cheryl McEwen
2011 – Robert Klein

ISSCR Tobias Award Lecture (2016-2021)

The ISSCR Tobias Award Lecture was established in 2015 by the Tobias Foundation. The award recognized original and promising basic hematology research as well as direct translational or clinical research related to cell therapy in hematological disorders.

Awardees:
2021 – Stuart H. Orkin
2020 – Margaret A. Goodell
2019 – Scott Armstrong
2018 – Connie Eaves
2017 – John Dick
2016 – Leonard Zon

ISSCR Award for Innovation (2018-2019; 2011-2017 called the McEwen Award for Innovation)

The ISSCR Award for Innovation recognized a transformative breakthrough that has had a major impact on the field of stem cell research or regenerative medicine.

Awardees:
2019 - John Dick
2018 – Michele De Luca, Graziella Pellegrini
2017 – Elaine Fuchs
2016 – Austin Smith, Qi-Long Ying
2015 – Hans Clevers, Irving Weissman
2014 – Azim Surani
2013 – James A. Thomson
2012 – Rudolf Jaenisch
2011 – Kazutoshi Takahaski, Shinya Yamanaka

Other awards:

The ISSCR Zhongmei Chen Yong Awards for Scientific Excellence (travel awards)
ISSCR Poster Awards
ISSCR Merit Abstract Awards

Supplementary Figure 2:
A list of ISSCR Awards and Awardees.
